# Diarylheptanoid 35d overcomes EGFR TKI resistance by inducing hsp70-mediated lysosomal degradation of EGFR in EGFR-mutant lung adenocarcinoma

**DOI:** 10.1016/j.jbc.2023.104814

**Published:** 2023-05-11

**Authors:** Xuan Hong, Min-Tsang Hsieh, Tzu-Yu Tseng, Hui-Yi Lin, Hung-Chih Chang, Sir-Theng Yau, Wei-Chung Cheng, Baozhen Ke, Hsiao-Hui Liao, Chih-Ying Wu, An-An Liu, Meei-Maan Wu, Kuo-Yen Huang, Pan-Chyr Yang, Sheng-Chu Kuo, Mien-Chie Hung, Pei-Chih Lee

**Affiliations:** 1 Department of Medical Oncology, Harbin Medical University Cancer Hospital, Harbin, China; 2 School of Pharmacy, China Medical University, Taichung, Taiwan; 3 Research Center for Chinese Herbal Medicine, China Medical University, Taichung, Taiwan; 4 Chinese Medicinal Research and Development Center, China Medical University Hospital, Taichung, Taiwan; 5 Research Center for Cancer Biology, China Medical University, Taichung, Taiwan; 6 Graduate Institute of Biomedical Sciences, China Medical University, Taichung, Taiwan; 7 Ph.D. Program for Cancer Molecular Biology and Drug Discovery, China Medical University, Taichung, Taiwan; 8 Department of Molecular and Cellular Oncology, The University of Texas MD Anderson Cancer Center, Houston, Texas, USA; 9 Department of Pathology and Laboratory Medicine, Taichung Veterans General Hospital, Taichung, Taiwan; 10 Department of Public Health, School of Medicine, College of Medicine, Taipei Medical University, Taipei, Taiwan; 11 School of Public Health, College of Public Health, Taipei Medical University, Taipei, Taiwan; 12 Master Program in Applied Epidemiology, College of Public Health, Taipei Medical University, Taipei, Taiwan; 13 Institute of Biomedical Sciences, Academia Sinica, Taipei, Taiwan; 14 Department of Internal Medicine, National Taiwan University Hospital and National Taiwan University College of Medicine, Taipei, Taiwan; 15 Genomics Research Center, Academia Sinica, Taipei, Taiwan

**Keywords:** EGFR, TKI-insensitive EGFR signaling, TKI resistance, lung adenocarcinoma, EGFR mutation, lung cancer

## Abstract

Epidermal growth factor receptor (EGFR)-mutant lung adenocarcinoma (LUAD) patients often respond to EGFR tyrosine kinase inhibitors (TKIs) initially but eventually develop resistance to TKIs. The switch of EGFR downstream signaling from TKI-sensitive to TKI-insensitive is a critical mechanism-driving resistance to TKIs. Identification of potential therapies to target EGFR effectively is a potential strategy to treat TKI-resistant LUADs. In this study, we developed a small molecule diarylheptanoid 35d, a curcumin derivative, that effectively suppressed EGFR protein expression, killed multiple TKI-resistant LUAD cells *in vitro*, and suppressed tumor growth of EGFR-mutant LUAD xenografts with variant TKI-resistant mechanisms including EGFR C797S mutations *in vivo*. Mechanically, 35d triggers heat shock protein 70–mediated lysosomal pathway through transcriptional activation of several components in the pathway, such as HSPA1B, to induce EGFR protein degradation. Interestingly, higher HSPA1B expression in LUAD tumors associated with longer survival of EGFR-mutant, TKI-treated patients, suggesting the role of HSPA1B on retarding TKI resistance and providing a rationale for combining 35d with EGFR TKIs. Our data showed that combination of 35d significantly inhibits tumor reprogression on osimertinib and prolongs mice survival. Overall, our results suggest 35d as a promising lead compound to suppress EGFR expression and provide important insights into the development of combination therapies for TKI-resistant LUADs, which could have translational potential for the treatment of this deadly disease.

Lung cancer is the leading cause of cancer-related deaths worldwide (1), with non–small cell lung cancers (NSCLCs) accounting for around 85 to 90% of cases. Of these NSCLCs, lung adenocarcinomas (LUADs) and lung squamous cell carcinoma (LUSC) make up ∼55% and ∼35%, respectively. Genetic mutation on epidermal growth factor receptor (EGFR) causes aberrant kinase activation in ∼50% of LUADs in Asian patients and ∼15% in Western population (2, 3). EGFR is known to play a critical role in maintaining tumor cell survival through its downstream signaling that rely on its kinase activity (4, 5, 6). Tyrosine kinase inhibitors (TKIs) have been effective in treating EGFR-mutant patients, but resistance develops after 10 to 18 months, and recurrent diseases currently lack effective therapies.

Several TKI-resistant mechanisms have been identified in EGFR-mutant NSCLCs, including upregulation of other receptor tyrosine kinases (RTKs) (7, 8) and development of additional EGFR mutations, such as C797S (9, 10, 11). Interestingly, the mechanisms of RTK-mediated TKI resistance have been linked to EGFR protein expression *per se*, independent of EGFR kinase activity (12). Upon TKI treatment, the upregulated RTKs in resistant tumors form heterodimers with TKI-inactive EGFR and activate downstream survival signaling (hereafter referred to as TKI-insensitive EGFR signaling). The TKI-insensitive EGFR signaling dominate the tumors upon TKI and contribute to heterogeneous TKI resistance and tumorigenesis (12, 13). Additionally, the acquisition of EGFR C797S mutations after TKI treatment also results in currently untreatable disease (7). Despite this, C797S-positive tumors may still rely on EGFR kinase activity for survival, as evidenced by their sensitivity to next-generation EGFR kinase inhibitors in preclinical models (10, 14). However, patients with C797S tumors still lack effective treatment in clinical. Therefore, pharmacological inhibitions of the TKI-insensitive EGFR signaling and the EGFR C797S by reducing EGFR protein expression may offer therapeutic opportunities for NSCLC patients with acquired TKI resistance.

Here, we developed a small molecule 35d which activates heat shock protein (hsp) 70–mediated lysosome pathway, degrading EGFR protein, and inhibiting TKI-insensitive EGFR signaling. 35d alone or with TKIs suppressed tumor growth in TKI-resistant LUAD tumors, including those with variant resistant mechanisms such as a common factor, nuclear PKCδ (found in >40% of the resistant LUADs) (12), and EGFR C797S mutation (found in ∼15% of the resistant LUADs) (7). These findings suggest that 35d could be a potential therapy for EGFR-mutant LUADs resistant to TKI.

## Results

### EGFR expression has clinical relevance in LUADs, but not LUSCs

**Figure 1:**
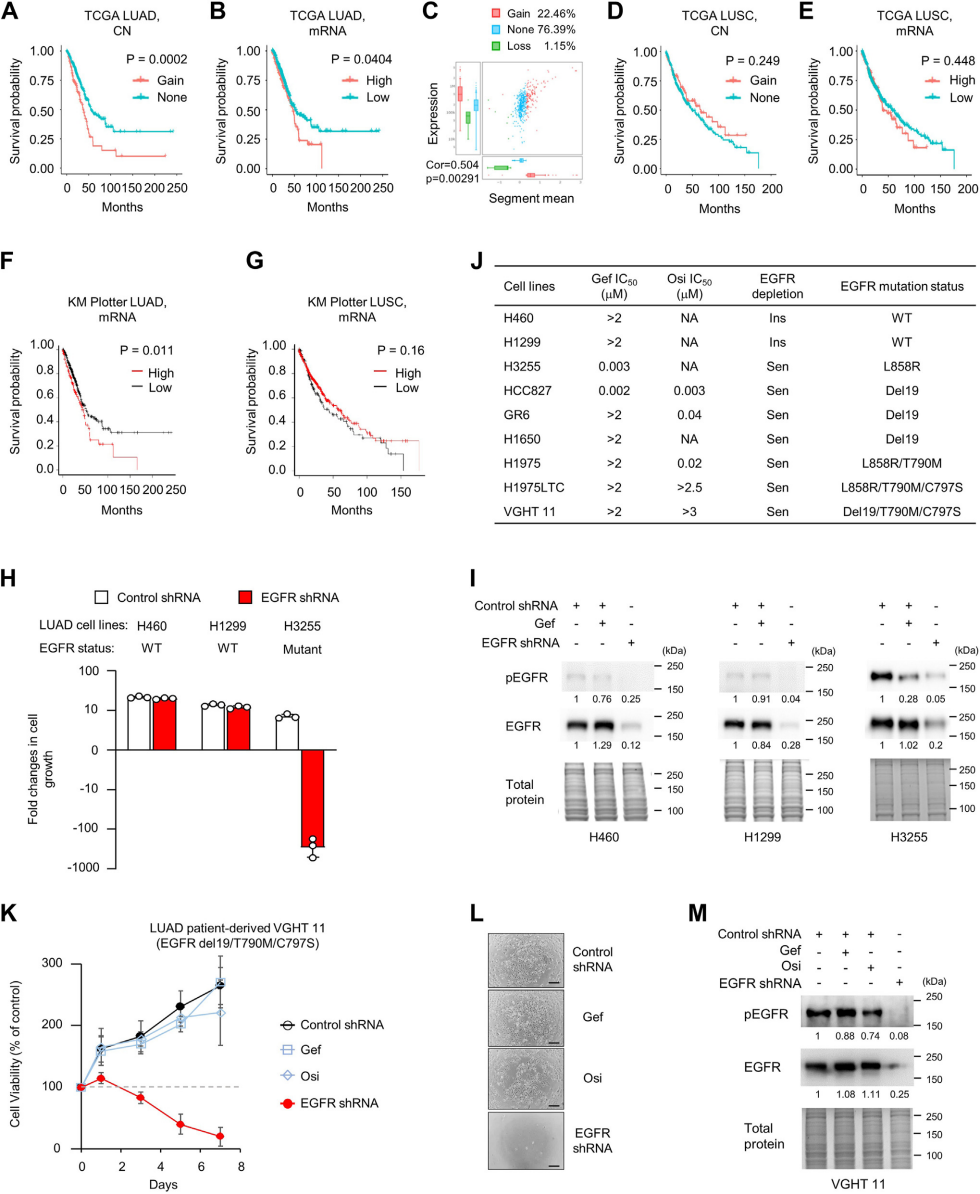
**Clinical relevance of EGFR expression in NSCLCs and TKI-insensitive roles of EGFR in EGFR-mutant TKI-resistant LUAD cells.***A*–*E*, EGFR DNA copy number, mRNA expression, and patient overall survival data for lung adenocarcinomas (LUADs) and lung squamous cell carcinoma (LUSC) from TCGA database. *A* and *D*, overall survival curves in LUAD patients with (n = 111) and without (n = 376) EGFR CN gain (*A*) and in LUSC patients with (n = 102) and without (n = 375) EGFR CN gain (*D*). *B* and *E*, overall survival curves for LUAD patients divided by the median value into low (n = 355) and high (n = 138) EGFR mRNA expression (*B*) and for LUSC patients divided by the median value into low (n = 96) and high (n = 398) EGFR mRNA expression (*E*). *C*, correlation of EGFR mRNA expression levels with EGFR CN in LUAD cohorts. *F* and *G*, EGFR mRNA expression and patient overall survival data for LUADs (*F*) and LUSCs (*G*) from KM plotter mRNA RNA-seq database. Overall survival curves for LUAD patients divided by the median value into low (n = 371) and high (n = 133) EGFR mRNA expression and for LUSC patients divided by the median value into low (n = 140) and high (n = 355) EGFR mRNA expression. *H*, EGFR WT H460, H1299 cells, and EGFR-mutant H3255 were counted after treatment of EGFR shRNA for 7 days. *I*, Western blot analysis of phosphorylated and total EGFR in EGFR shRNA cells and in control shRNA cells treated with 1 μM gefitinib (gef). *J*, IC_50_ of EGFR TKI gef and osimertinib (osi), as well as cell response to EGFR shRNA (EGFR depletion) in LUAD cells with various EGFR mutations. *K*–*M*, responses to TKIs and EGFR shRNA in patient-derived VGHT 11 cells harboring EGFR del19/T790M/C797S mutations. VGHT 11 cells were infected with control or EGFR shRNA. The control shRNA cells were then treated with 0.1 μM gef and 0.1 μM osi. The cells were counted after treatment at the indicated time points (*K*). Representative bright-field microscopy images of VGHT 11 cells treated as in (*K*) at day 7 (*L*). The scale bar represents 500 μm. *M*, Western blot analysis of phosphorylated and total EGFR in EGFR shRNA cells and in control shRNA cells treated with gef or osi for 24 h. Relative pEGFR and EGFR levels are indicated below the blot. Total protein was used as the loading control. Molecular weight markers are noted next to all immunoblots. *I* and *K*, data are presented as mean ± SD (n = 3). CN, copy number; EGFR, epidermal growth factor receptor; Ins, insensitive; NSCLC, non–small cell lung cancer; Sen, sensitive; TKI, tyrosine kinase inhibitor.

EGFR, an important RTK and oncogene driver, plays a vital role in NSCLC (15). However, inhibition of EGFR kinase activity by clinical TKIs only benefits to a subpopulation of NSCLC patients (16, 17). To systematically investigate whether TKI-insensitive EGFR signaling is critical for NSCLCs, we analyzed the EGFR expression by evaluating DNA copy number (CN) and mRNA expression levels in LUAD and LUSC cohorts from The Cancer Genome Atlas (TCGA) NSCLC dataset. We also determined whether EGFR CN and mRNA expression levels can predict patient survival. The patients with EGFR CN gain exhibited shorter overall survival (OS) than the patients with normal CN in LUAD subset (Fig. 1*A*, *p* = 0.0002). Consistently, higher EGFR mRNA expression in the tumors was associated with inferior OS of LUAD patients (Fig. 1*B*, *p* = 0.0404). Furthermore, a significantly positive correlation between EGFR gene CN and mRNA expression levels were found in LUAD tumors (Fig. 1*C*, *p* = 0.00291). However, in LUSC cohort, there were no correlation of EGFR CN alteration (Fig. 1*D*, *p* = 0.249) and mRNA levels (Fig. 1*E*, *p* = 0.448) with patient OS. The results were subsequently confirmed using Kaplan–Meier plotter mRNA RNA-seq dataset (18), where high EGFR mRNA expression associated with poor OS in LUAD (Fig. 1*F*, *p* = 0.011), but not in LUSC (Fig. 1*G*, *p* = 0.16). These data suggested that EGFR expression levels in the tumors are critical for patient survival in LUAD, but not in LUSC.

### Cell-based analysis revealed TKI-insensitive role of EGFR in EGFR-mutant, TKI-resistant LUAD cells, but not in EGFR WT cells

While EGFR mutation status is commonly used as a therapeutic marker for TKIs in treating lung cancer, our findings indicated that the expression levels of EGFR in tumors are still critical for survival, especially in LUAD patients, suggesting a potential role of TKI-insensitive EGFR signaling in LUADs. Therefore, we investigated whether EGFR status determines the dependence of LUADs on TKI-insensitive EGFR signaling. Several LUAD cell lines with variant EGFR status and TKI response were treated with EGFR shRNA to block TKI-insensitive EGFR signaling through depleting EGFR expression (Fig. 1, *H*–*M*). Cell viability analysis showed that EGFR-WT H460 and H1299 cells were insensitive to both EGFR kinase inhibitor gefitinib (gef) and EGFR shRNA (Fig. 1, *H* and *I*), suggesting that TKI-insensitive EGFR signaling may be not essential for survival of these EGFR-WT LUAD cell lines. On the contrary, EGFR L858R-mutant H3255 cells highly sensitized to EGFR shRNA (Fig. 1, *H* and *I*), which are expected because the H3255 cells harboring EGFR-activating mutation sensitize to EGFR inhibitor. The EGFR additional C797S mutation was currently discovered as one of the major acquired resistance mechanisms to the third generation TKI, osimertinib (osi) (19), and such patients do not have any effective treatment in clinical. We next tested the effects of EGFR depletion in a patient-derived VGHT 11 cells harboring del19/T790M/C797S mutations on EGFR (20). We confirmed that the T790M/C797S-positive VGHT 11 cells were resistant to both first generation TKI gef and third generation TKI osi (Fig. 1, *K* and *L*). Importantly, we found that the cell growth of VGHT 11 cells was markedly suppressed by the treatment of EGFR shRNA (Fig. 1, *K*–*M*), suggesting a therapeutic potential for the untreatable C797S-mutant tumors. Previous study reported that EGFR-mutant H1650 cells that harbor intrinsically resistant mechanisms to TKIs through activation of PKCδ, AKT, and NF-kB pathways were susceptible to EGFR depletion (12, 21, 22). All these results indicated that EGFR-mutant cells, but not EGFR WT cells we tested, may sensitize to EGFR depletion and suggested that targeting of TKI-insensitive EGFR signaling by reducing EGFR protein expression may be a therapeutic strategy for treating TKI-insensitive or TKI-acquired resistant patients with EGFR-mutant LUADs, which patients currently lack effective treatment in clinical.

### Text-mining analysis, experimental chemical modifications of the lead compound, and cell-based growth-inhibitory drug screenings unveiled anticancer potentials of a small molecule 35d for treating EGFR-mutant, TKI-resistant LUADs

**Figure 2:**
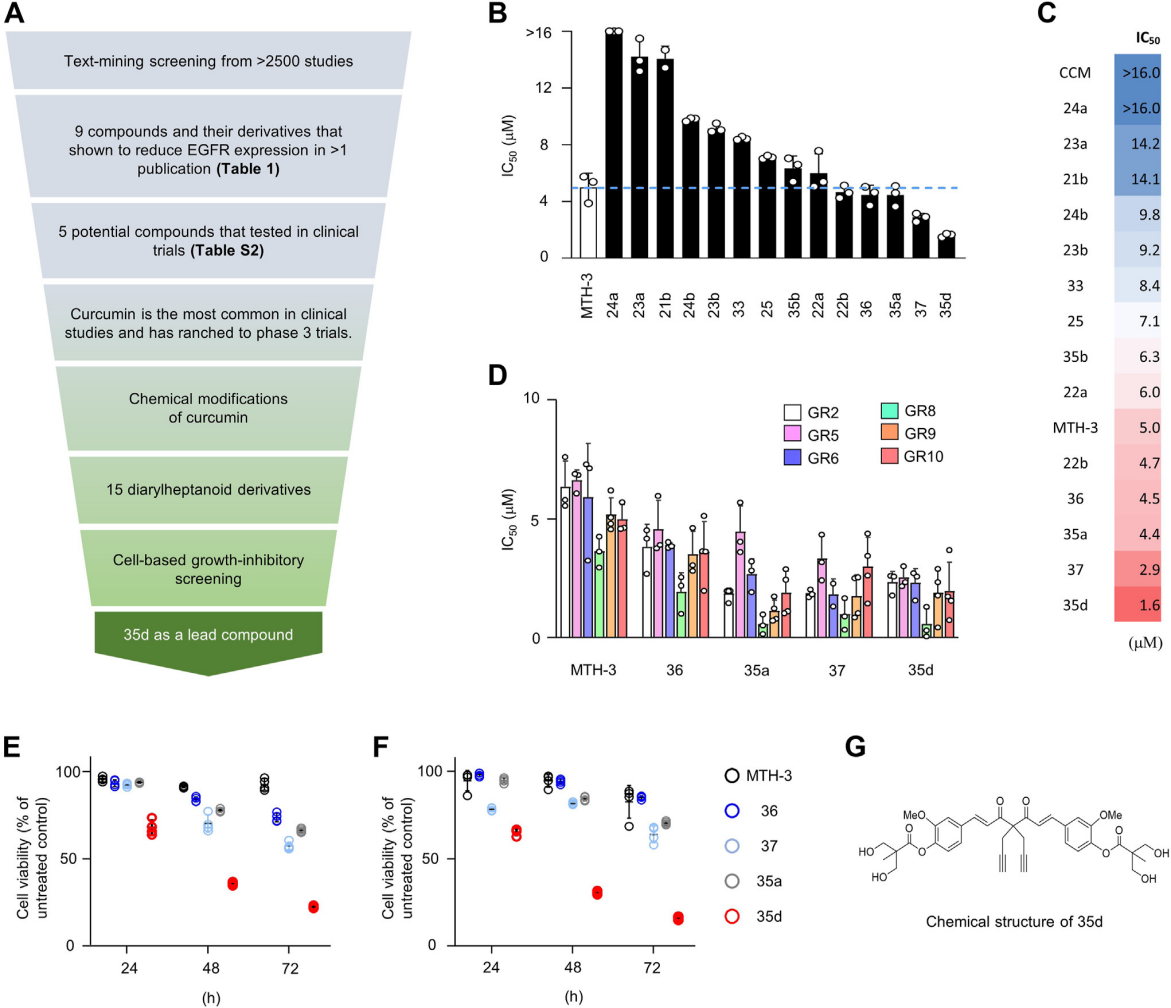
**Development of potential curcumin derivative 35d as anticancer agents for EGFR-mutant TKI-resistant LUAD cells.***A*, schematic description of screening strategies for identifying and developing potential compounds for treating EGFR-mutant TKI-resistant LUAD tumors. *B*–*D*, bar graphs (*B* and *D*) and heat map (*C*) depicting IC_50_ value of curcumin (CCM) derivatives from *in vitro* cell viability assays in EGFR-mutant TKI-resistant LUAD H1650 cells (*B* and *C*) and six acquired gef-resistant (GR) clones (*D*). The cells were treated with indicated compounds for 5 days and subjected to cell viability assay by MTT analysis. IC_50_ >16 μM indicates cells treated with up to 16 μM of indicated compounds did not reach 50% of cell growth inhibition in H1650 cells. *E* and *F*, cytotoxicity assay of MTH-3, 36, 35a, 37, and 35d in H1650 (*E*) as well as EGFR L858R/T790M mutant H1975 cells (*F*). The cells were treated with indicated compounds (2 μM) for 24, 48, and 72 h. Cell viability were assessed by MTT assay and normalized to vehicle-treated control at each time point. Data are presented as mean ± SD (n = 3). *G*, chemical structure of diarylheptanoid 35d. EGFR, epidermal growth factor receptor; LUAD, lung adenocarcinoma; MTT, 3-[4,5-dimethylthiazol2-yl]-2,5-diphenyltetrazolium bromide; TKI, tyrosine kinase inhibitor.

To convert the *in vitro* concept to practical practices in preclinical and in clinical, we looked for emerged candidate agents that exerted to reduce EGFR expression and have potential to further develop as anticancer drugs. We tended to focus on natural compounds as many of these compounds were generally tolerable in human. Text-mining screenings were performed to identify compound candidates that were showed to reduce EGFR expression in which we searched more than 2500 preclinical and clinical studies between 1995 and 2021 (Fig. 2*A*). Nine compound candidates were identified, which have been showed to reduce EGFR expression in cancer cells and were reproduced in at least two literatures (Fig. 2*A*, Tables 1 and S1). Further investigation of these compounds indicated that five of them have been tested in clinical trials (Fig. 2*A* and Table S2). Among these candidates, curcumin has been shown to reduce EGFR expression in several studies (Fig. 2*A* and Table 1) and has been used to treat malignant diseases, including lung cancers (23, 24). Moreover, curcumin has been tested in more than 270 clinical trials including more than 25 phase 3 trials with well-tolerant doses (Table S2), indicating that curcumin is a highly safe agent in human. However, poor chemical stability, low water solubility, and low oral bioavailability remain major challenges for curcumin in the utilization as a therapeutic drug (25). We have previously modified and synthesized several series of diarylheptanoid derivatives based on chemical structure of curcumin (26). Among the derivatives, MTH-3 was the first to be synthesized and showed greater cell-growth inhibitory activities than curcumin against breast cancer cells (27). To evaluate whether these diarylheptanoid derivatives suppress EGFR expression and inhibit the tumor growth in distinct EGFR-mutant, TKI-resistant lung cancer models, we performed a growth inhibitory screening by treating curcumin, MTH-3, and 14 diarylheptanoid derivatives in EGFR-mutant H1650 cells (Fig. 2, *B* and *C*), which cells are known to be intrinsically resistant to EGFR kinase inhibition but sensitize to EGFR protein depletion (12). We found that among the 14 diarylheptanoid derivatives, 22b, 36, 35a, 37, and 35d showed lower IC_50_ values than MTH-3 and curcumin, indicating their greater effects in killing H1650 cells (Fig. 2, *B* and *C*). Among these five derivatives, 22b showed relatively low solubility (data not shown), which may cause failure for drug development in the future, and was excluded for following analysis.

**Table 1:** Emerged natural compounds that reduce EGFR expression in cancer cells

Compound	Types of cancer cells	Detection methods
Curcumin and its derivatives	Bladder: 253JB-V, KU7 Breast: MDA-MB-231 Colon: RKO, SW480 Lung: CL1-5, A549, H1975; H226 Nasopharyngeal: CNE-2 Osteosarcoma: MG-63 Pancreas: P34, PC14 Skin: A431	WB
Cucurbitacin B	Breast: MDA-MB-231, SKBR3 Cholangiocarcinoma: KKU-100 Pancreatic: BxPC3, HPAC	WB
Deguelin	Breast cancer: MDA-MB-231 HNSCC: SCC-4	WB, IF
(−)-Epigallocatechin-3-gallate	Breast: MDA-MB-231, MCF-7 Bronchial: BEAS-2B Colorectal: Caco-2 Esophageal: KYSE150, A431 Head and neck: SACC-83 Lung: A549	WB
Honokiol/liposomal honokiol	Brain: U251, U87MG Bronchial: 1117 Head and neck: FaDu, SCC-1, Cal-33, KB-3-1 Liver: Hep3B, HepG2 Lung: PC-9, H1975, HCC827, H460, SPC-A1	WB, IF
Plumbagin and its derivative	Lung: H460 Pancreas: PANC1, BxPC3, ASPC1	WB
Quercetin/gold nanoparticles-conjugated quercetin	Breast: MDA-MB-231, MCF-7 Cervical: HeLa Prostate: PC-3, LNCaP	WB
Resveratrol	Esophageal cancer: KYSE150, Eca109 Glioma: C6	WB
Sulforaphane	Breast cancer: MDA-MB-231, MDA-MB-468, T47D Lung cancer: A549, H1975, H3255, PC9	WB

The compounds in this table were showed to reduce EGFR expression in more than one publication.

Abbreviation: IF, immunofluorescence analysis.

We have modeled acquired resistance to gef from TKI-sensitive, EGFR-mutant LUAD HCC827 cells and followed by a single cell isolation (12). Several gef-resistant (GR) clones have been generated, and all the GR clones are highly resistant to gef and afatinib compared to their parental cells. Each GR clone expresses several and distinguished resistant mechanisms independently of EGFR T790M mutations (12). In this study, we evaluated anticancer effects of the four derivatives in our acquired resistance models. The results indicated that all the GR cells were more sensitive to these four diarylheptanoid derivatives than MTH-3 (Fig. 2*D*). To further evaluate the effects of these compounds, cytotoxicity analysis of the four diarylheptanoid derivatives and MTH-3 were performed in H1650 and in a well-characterized gef-resistant H1975 cell model with EGFR T790M mutation (Fig. 2, *E* and *F*, respectively). Among the four derivatives, 35d (Fig. 2*G*) is the most effective to inhibit cell growth in H1650 and H1975 cells. Our data from intrinsically TKI-resistant cell line, acquired resistant clones, and T790M-positive cell line suggest therapeutic potential of 35d for these clinically irremediable LUADs. Moreover, we noticed that the IC_50_ of curcumin, MTH-3, and 35d are dropping from >16, 3.6 ∼ 6.6, to 0.5 ∼ 2.5 μM, respectively, in all the TKI-resistant LUAD cells we tested (Fig. 2*C*), indicating that our chemical modification strategies effectively improve their anticancer activities against EGFR-mutant, TKI-resistant LUAD cells.

### Global transcriptome analysis of 35d-treated cells uncovered specific upregulation of heat shock protein 70 pathways in EGFR-mutant, TKI-resistant LUAD cells

**Figure 3:**
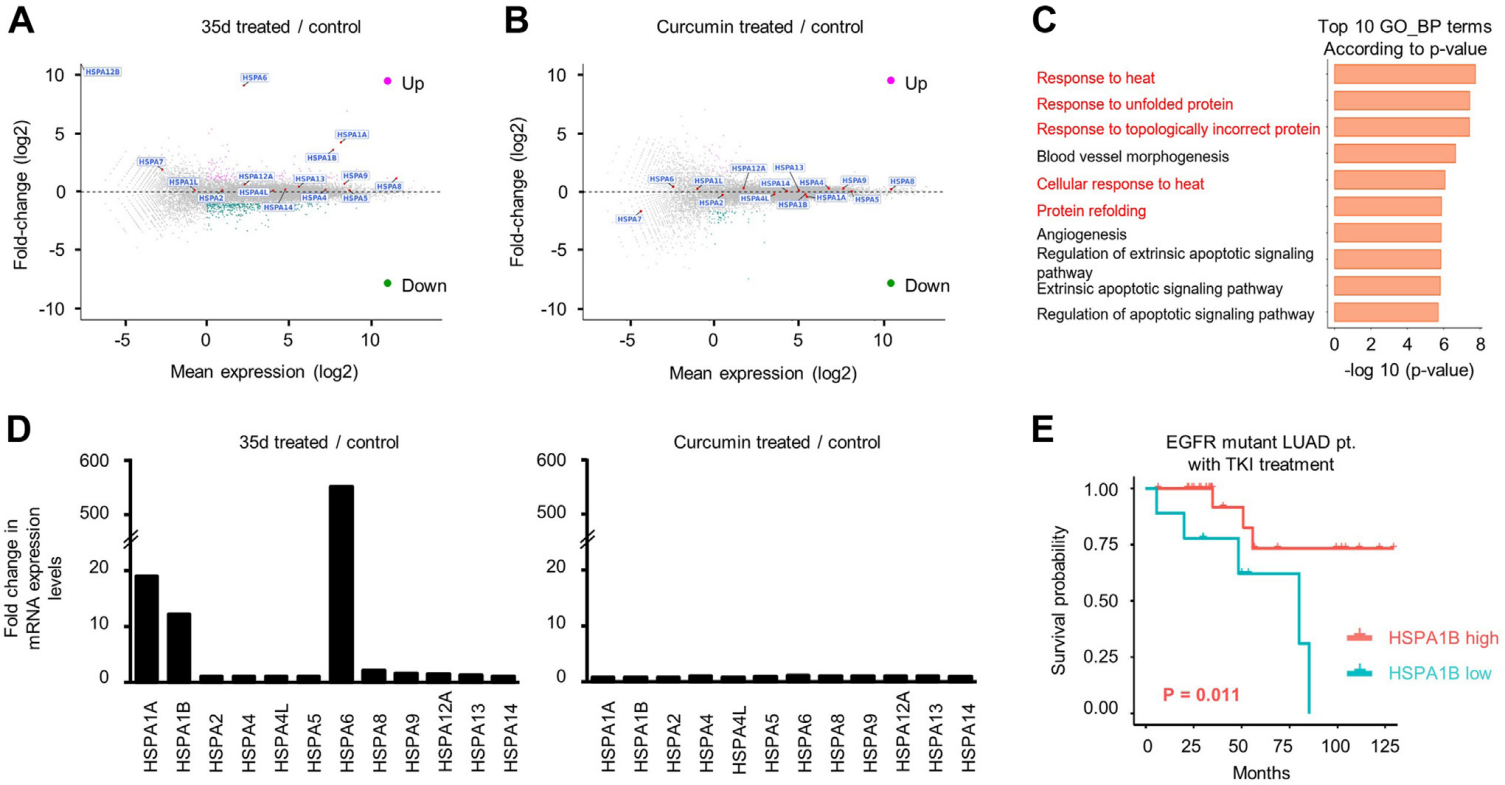
**Transcriptome profile of RNA seq in 35d-treated cells and clinical survival analysis of HSPA1B expression in LUAD cohorts.***A* and *B*, MA plot showing genes that are differentially expressed (log2 fold change >1 (*up*) and <−1 (*down*)) in RNA-seq data between 35d-treated cells (*A*) and curcumin-treated cells (*B*) *versus* control-treated cells. RNA-seq analysis was performed in GR6 cells treated with control, 35d (1 μM), and curcumin (1 μM) for 24 h. *C*, 35d treatment underwent transcriptomic changes toward increased heat shock response. Shown are gene set enrichment analyses of RNA-seq data as described in (*A*) using gene set annotated in the Gene Ontology (GO). Functions related to heat shock response are labeled in *red*. *D*, transcriptional changes of 12 heat shock protein 70 family genes in 35d (*left*)- and curcumin (*right*)-treated GR6 cells based on RNA-seq data from (*A*) and (*B*), respectively. *E*, effects of HSPA1B on overall survival in East Asia LUAD cohort (28). Kaplan–Meier survival curves for EGFR-mutant patients with TKI treatment divided into low and high HSPA1B mRNA expression. EGFR, epidermal growth factor receptor; GR, gef-resistant; HSP, heat shock protein; LUAD, lung adenocarcinoma; TKI, tyrosine kinase inhibitor.

To explore the specific mechanisms in TKI-resistant cells treated with 35d, globally transcriptional analysis by RNA-seq was performed in 35d- and curcumin-treated GR6 cells (Fig. 3, *A* and *B*). Principal component analyses of the RNA-seq data revealed global changes by 35d treatment in the transcriptome (Fig. 3*C*). Interestingly, five of top ten gene sets were related to aspects of heat responses, such as response to heat, response to unfolded protein, and cellular response to heat (Fig. 3*C*, red). Three of them were apoptotic signaling pathways, suggesting that 35d may induce cancer cell death through apoptosis (Fig. 3*C*). To identify the molecular events that trigger the heat responses in the 35d-treated cells, we revisited our RNA-seq data. We found that several HSP70 family genes were involved in the heat response gene sets (Table S3). Further analysis of all HSP70 family genes in the RNA-seq data revealed that mRNA expression levels of HSPA1A, HSPA1B, HSPA6, and HSPA8 were >2-fold increased in response to 35d (Fig. 3*D*, left). Interestingly, curcumin at the same concentration did not affect any of these HSP70 gene expressions (Fig. 3*D* right). To investigate the clinical significance of the HSP70 family genes in LUADs, we analyzed a human LUAD dataset, containing crucial patient information such as tumor EGFR mutation status, gene expression profile, patient TKI-treatment history, and OS (28). Using this dataset, we categorized the LUAD patients into three cohorts based on EGFR status and TKI treatment (EGFR-mutant patients with TKI treatment, EGFR-mutant patients without TKI treatment, and EGFR WT patients without TKI treatment). We then correlated the mRNA expression levels of all the HSP70 family genes with patient OS in each cohort (Table S4). Importantly, among the 15 HSP70 family genes analyzed, our results revealed that higher HSPA1B significantly correlated with longer OS in the EGFR-mutant patients with TKI treatment (*p* = 0.011; Table S4 and Fig. 3*E*). However, this correlation was not observed in the EGFR-mutant patients without TKI and the EGFR WT patients (*p* = 0.998 and 0.964, respectively; Table S4). These results indicated that 35d upregulated HSP70 genes in the EGFR-mutant, TKI-resistant cells and suggested high HSPA1B expression in the tumors may be clinically beneficial for only the EGFR-mutant LUAD patients who underwent TKI treatment.

### Diarylheptanoid 35d effectively suppresses EGFR expression

**Figure 4:**
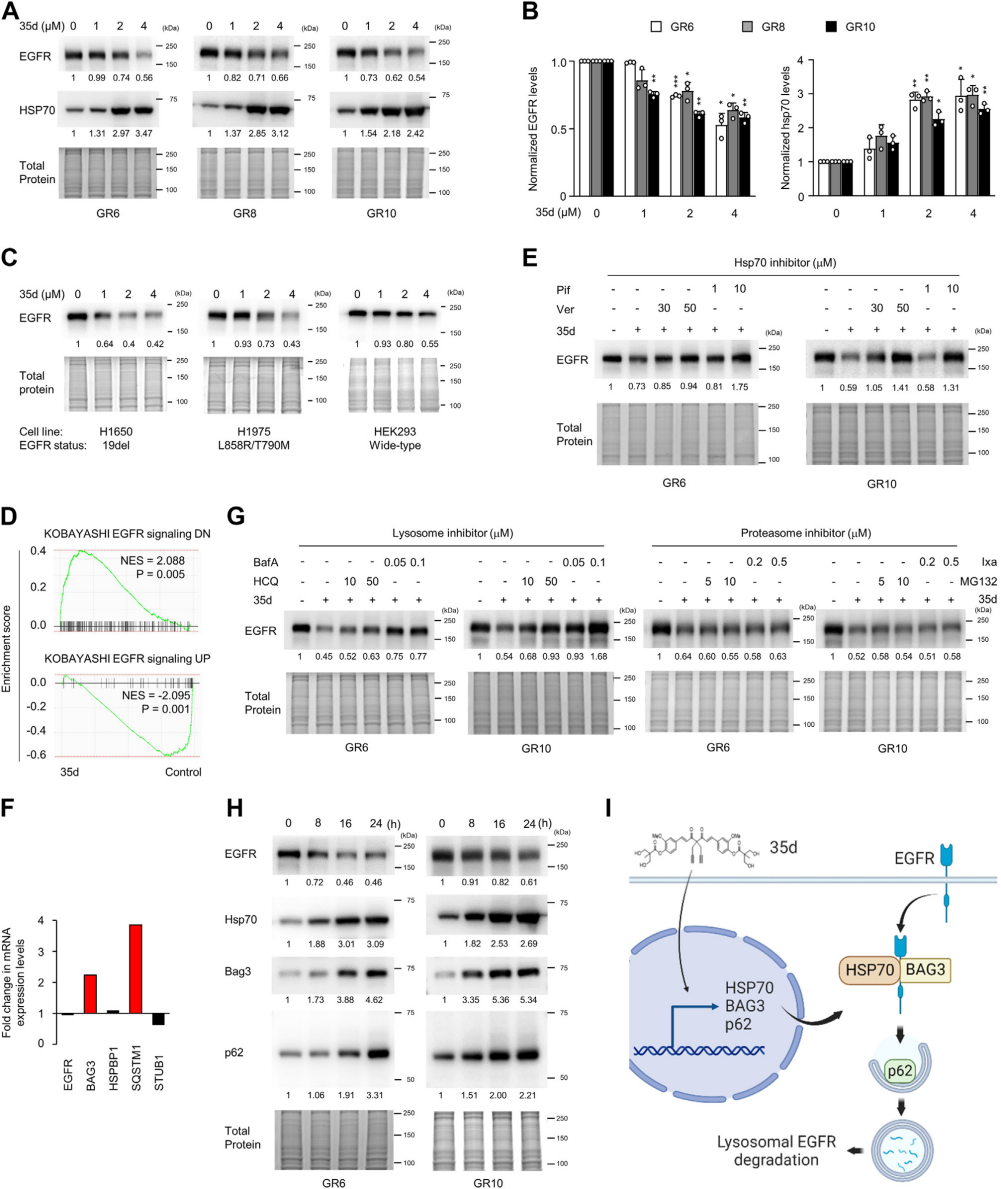
**Inhibition of EGFR expression and downstream signaling by 35d through hsp70-mediated lysosomal degradation in EGFR-mutant LUAD tumors with TKI** r**esistance**. *A* and *B*, EGFR and HSP70 expression in GR6, GR8, and GR10 cells treated with 35d for 24 h were determined by Western blot analysis (*A*). *B*, quantitation of EGFR and HSP70 expression in GR6, GR8, and GR10 cells from (*A*). Results are expressed relative to total protein. Comparisons between each treatment group with its untreated control group were analyzed using Student’s *t* test followed by the Bonferroni correction for three comparisons. ∗*p* < 0.05, ∗∗*p* < 0.01. *C*, EGFR expression in H1650, H1975, and HEK293 cells treated with 35d for 24 h were determined by Western blot analysis. *D*, GSEA of RNA-seq data as described in Figure 3*A* using KOBAYASHI EGFR signaling DN and UP pathway gene sets. *E*, Western blot showing EGFR expression in GR6 and GR10 cells treated with 35d (2 μM) and hsp70 inhibitor ver-155008 (ver) and pifithrin (pif) at the indicated concentrations (μM) for 24 h. *F*, fold changes in EGFR, BAG3, HSPBP1, SQSTM1, and STUB1 mRNA levels in GR6 cells treated with 35d in RNA-seq data as described in Figure 3*A*. *G*, Western blot showing EGFR expression in GR6 and GR10 cells treated with 35d (2 μM) and lysosome inhibitors hydroxychloroquine (HCQ), bafilomycinA (bafA) and proteasome inhibitors MG132, ixazomib (ixa) at the indicated concentrations for 24 h. *H*, protein expression of EGFR, hsp70, bag3, and p62 analyzed at the indicated time points in GR6 and GR10 cells treated with 35d (2 μM). Relative protein levels are indicated below the blot. Total protein was used as the loading control. Molecular weight markers are noted next to all immunoblots. Quantitation data are from three independent experiments and presented as mean ± SD (*B*). *I*, model depicting EGFR protein degradation in response to 35d treatment. EGFR, epidermal growth factor receptor; GR, gef-resistant; GSEA, gene set enrichment analysis; HSP, heat shock protein; LUAD, lung adenocarcinoma; TKI, tyrosine kinase inhibitor.

Our data indicate that the EGFR-mutant LUAD with TKI resistance is a unique subtype among NSCLCs that relies on the EGFR protein expression *per se* for survival (Fig. 1). Our data has shown that a synthesized, small molecule curcumin derivative 35d is highly effective in inhibiting the growth of these cells (Fig. 2). To better understand how 35d achieves these effects, we investigated whether it works by reducing EGFR expression. The result indicated that 35d dose dependently reduced EGFR protein expression in various EGFR-mutant, TKI-resistant cells including GR6, GR8, GR10 (Fig. 4, *A* and *B* left), H1650, H1975 (Fig. 4*C*), and in EGFR WT HEK293 cells (Fig. 4*C*, right). On the other hand, curcumin at the same dose did not exhibit any effects on EGFR expression (Fig. S1). To assess the inhibitory effects of 35d on EGFR signaling in the resistant cells, we reanalyzed our RNA-seq data. Gene set enrichment analysis (GSEA) analyses indicated that EGFR signaling down (DN) profiles were increased and EGFR signaling up profiles were decreased upon 35d treatment in GR6 cells (Fig. 4*D*). Thus, 35d reduced EGFR expression and comprehensively inhibited the EGFR signaling pathway in TKI-resistant cells.

HSPA1B, encoding the hsp70, is a recognized molecular chaperone known to be involved in the targeting of oncogenic proteins for intracellular degradation (29). Our findings demonstrated that 35d upregulated HSPA1B gene expression and reduced EGFR protein expression. These data draw us to hypothesize that 35d may reduce EGFR expression by activating hsp70-mediated pathways. To test this, we first showed that hsp70 protein expression was substantially induced by 35d in GR6 and GR10 cells (Fig. 4, *A* and *B* right). Commercial hsp70 inhibitors pifithrin-μ (pif) and VER-155008 (ver) were then used to block hsp70-mediated pathways in EGFR-mutant, TKI-resistant cells. Inhibition of hsp70 by pif and ver reversed 35d-reduced EGFR expression in GR6 and GR10 cells (Fig. 4*E*), indicating that 35d led to suppression of EGFR protein through hsp70-mediated pathways. Hsp70 complex has been reported to involve lysosome or proteasome pathways as major degradation systems in the cells to regulate their client proteins (29, 30). Interestingly, our data showed that 35d reduced EGFR protein expression (Fig. 4*A*), but not mRNA expression (Fig. 4*F*), suggesting that 35d may lead to EGFR protein degradation through induction of hsp70 pathway. Next, we showed that lysosome inhibitors bafilomycin A1 and hydroxychloroquine, but not proteasome inhibitors ixazomib and MG132, significantly reversed the 35d-reduced EGFR protein expression (Fig. 4*G*). Therefore, treatment of 35d induced EGFR protein degradation through hsp70-mediated lysosome pathway. To investigate the expression changes of key components in the hsp70-mediated lysosome pathway in 35d-treated cells, we revisited our RNA-seq data in which the key component genes BAG3, HSPBP1, SQSTM1, and STUB1 were detectable in the assay. We found 2.24- and 3.85-fold increased expression of BAG3 (encoding bag3 protein) and SQSTM1 (encoding p62 protein) genes, respectively, in 35d-treated cells compared to untreated cells, but not HSPBP1 and STUB1 (Fig. 4*G*). Bag3, an instructed protein partner in the hsp70 complex, was reported to recruit p62, a lysosome receptor, to the chaperone–hsp70 complex leading to client protein degradation *via* the lysosome pathway (31, 32). Accordingly, 35d may specifically activate hsp70-mediated lysosome pathway through transcriptional activations of HSPA1B, BAG3, and SQSTM1 genes and in turn led to EGFR protein degradation in EGFR-mutant, TKI-resistant LUAD cells. To further dissect these dynamics, we collected cell lysates from GR6 and GR10 cells at different time points during 35d treatment and the lysates were subjected to Western blot (WB) analysis. Our data indicated that 35d-induced hsp70, bag3, and p62 expression were significantly found, while degradation of EGFR began after 16 h of treatment with 35d (Fig. 4*H*). Together, treatment of 35d upregulates the expression of hsp70 complex components, hsp70, bag3, and p62, which lead to EGFR protein degradation through lysosome-dependent pathway (Fig. 4*I*). All these data of mechanistic basis supported that 35d effectively suppressed EGFR expression and have potential to be an anticancer drug for EGFR-mutant, TKI-resistant LUADs.

### Anticancer effects of 35d in multiple TKI-resistant LUADs

**Figure 5:**
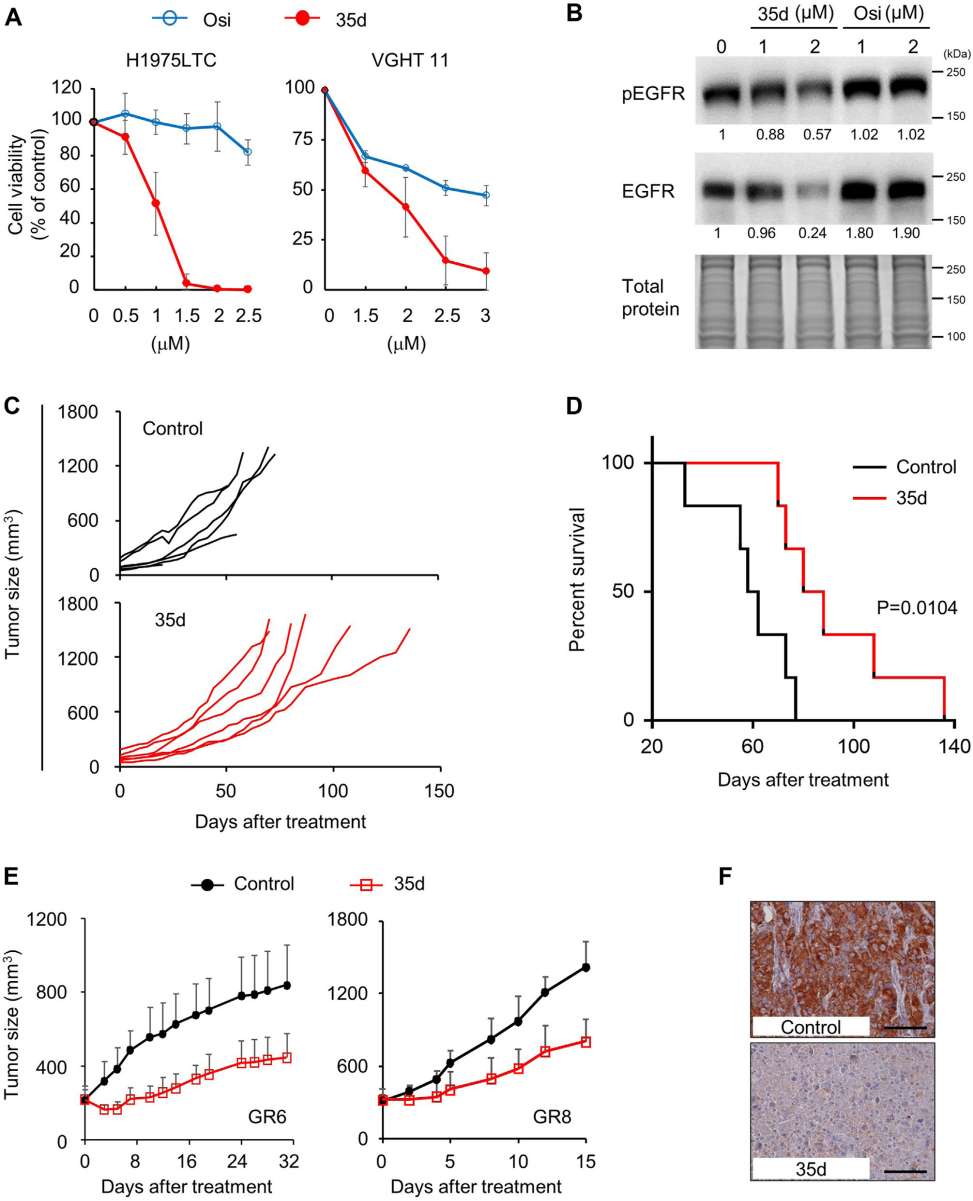
**Inhibition of tumorigenesis by 35d in EGFR-mutant LUAD tumors with TKI** r**esistance, including EGFR C797S.***A*, comparison of dose response to 35d and osimertinib (osi) in EGFR C797S–positive cells, H1975LTC (*left*), and patient-derived VGHT 11 (*right*). Cells were treated with 35d and osi for 6 days and subjected to cell viability assay by crystal violet staining. Data are represented as mean ± SD (n = 3). *B*, Western blot analysis of phosphorylated and total EGFR in VGHT 11 cells treated with 35d and osi for 24 h. Relative pEGFR and EGFR levels are indicated below the blot. Total protein was used as the loading control. Molecular weight markers are noted next to all immunoblots. *C*, tumor growth of VGHT 11 xenografts in mice treated with or without 35d. Established VGHT 11 tumors (n = 6 per group) were randomized and treated with 35d (100 mg/kg) 6 days per week followed by measurement of tumor volumes. Tumor size was measured with a caliper 2 times per week. *D*, Kaplan–Meier survival curves of VGHT 11 tumor-bearing mice treated with 35d (100 mg/kg). *E*, tumor growth of GR6 (*left*) and GR8 (*right*) xenografts in mice treated with or without 35d. Established tumors (250–300 mm^3^, n = 10) were randomized and treated with 35d (100 mg/kg) 6 days per week followed by measurement of tumor volumes. Tumor size was measured with a caliper 2 to 3 times per week. Data are represented as mean ± SD. *F*, immunohistochemistry of EGFR in the tumor sections from 35d-treated GR8 tumors at day 15 as in (*E*). The scale bar represents 100 μm. EGFR, epidermal growth factor receptor; GR, gef-resistant; LUAD, lung adenocarcinoma; TKI, tyrosine kinase inhibitor.

To examine the effects of 35d on the C797S-mediated TKI resistance, two C797S-positive LUAD cells (H1975LTC, a stable EGFR L858R/T790M/C797S-expressing H1975 cells and VGHT 11, a patient-derived cells harboring EGFR del19/T790M/C797S; Fig. 1*J*) were treated with 35d and osi (Fig. 5*A*). Remarkably, at concentrations of 1.5 to 2.5 μM, treatment of 35d resulted in complete suppression of cell growth in H1975LTC cells, whereas treatment of osi at the same concentrations only slightly affects the cell growth (Fig. 5*A*, left). Consistently, treatment of 35d effectively inhibited cell growth in the patient-derived VGHT 11 cells as compared to treatment of osi (Fig. 5*A*, right). Moreover, 35d is effective to reduce protein expression of C797S-mutant EGFR (Fig. 5*B*). To determine whether 35d is active against C797S-positive tumors *in vivo*, VGHT 11 xenograft mice were treated with vehicle or 35d. The results indicated that 35d inhibited VGHT 11 tumor growth (Fig. 5*C*) and significantly extended mice survival (Fig. 5*D*). These *in vitro* and *in vivo* data from cell lines, acquired resistant clones, and patient-derived cells indicated that 35d have potential to inhibit tumor growth in EGFR-mutant LUADs with distinguished TKI-resistance mechanisms, including EGFR C797S mutation.

To determine whether 35d is active against other TKI-resistant tumors *in vivo*, GR6 and GR8 xenograft mice were treated with vehicle or 35d at 100 mg/kg orally six times per week. 35d-treated mice showed reduced tumor volumes compared with control mice in the GR6 and GR8 bearing SCID (CB17-Prkdc^scid^/NcrCrl) mice models (Fig. 5*E*). Inhibition of GR6 and GR8 tumor growth occurred early and was sustained for 32 and 15 days, respectively, following 35d treatment. Immunohistochemistry of the tumor sections demonstrated that EGFR expressions were suppressed by 35d *in vivo* (Fig. 5*F*). These results indicated that treatment of 35d downregulated EGFR protein expression to block TKI-insensitive EGFR signaling and led to inhibition of tumorigenesis in EGFR-mutant, TKI-resistant LUADs.

### Combination of 35d with EGFR kinase inhibitor is an effective and safe treatment for EGFR-mutant, TKI-resistant LUADs

**Figure 6:**
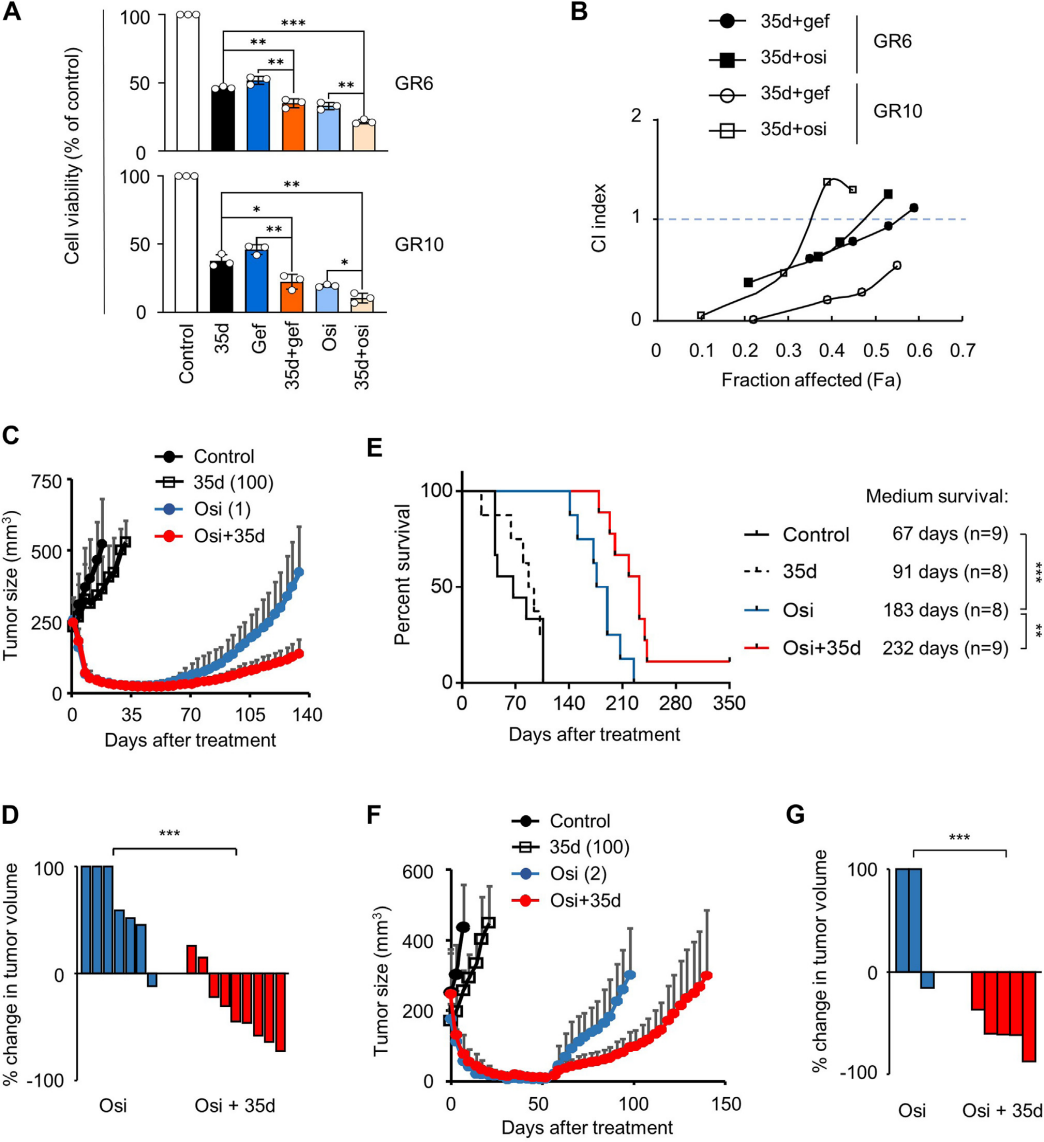
**Rationales of combined 35d and clinical TKIs in EGFR-mutant TKI-resistant LUAD tumors.***A* and *B*, combination effects of 35d and TKIs *in vitro*. GR6 and GR10 cells were treated with 35d (2.5 μM) in combination of gef (5 μM) and osi (5 μM) for 3 days. Cell viability was assayed as described in Figure 2*B* (*A*), and combination indexes (CI) were calculated (*B*). Comparisons between each combination treatment group (*i.e.*, 35d+gef and 35d+osi) with its single treatment group (*i.e.*, 35d alone, gef alone, and osi alone) were analyzed using Student’s *t* test to compare two groups of independent samples followed by the Bonferroni correction for two comparisons. ∗*p* < 0.05, ∗∗*p* < 0.01, ∗∗∗*p* < 0.001. CI < 1, synergy; CI = 1, additive; CI > 1, antagonism. *C*–*E*, the combination of 35d and osi delays the emergence of osi resistance in EGFR-mutant HCC827 tumor models. *C*, mice bearing HCC827 xenografts were treated with 35d (100 mg/kg), osi (1 mg/kg), or the combination six times per week when the tumor volume reached 200 to 250 mm^3^ (n = 7–9 per arm). Tumor size was measured with a caliper twice per week. *D*, a waterfall plot representation of the changes in tumor volume of each tumor taken at day 134 of the osi alone and the osi-35d combination treatment in the xenograft mice. Tumor volumes above 100% are truncated at 100%. *E*, Kaplan–Meier survival curves and medium survival of HCC827 tumor-bearing mice treated with 35d, osi, or the combination. *F* and *G*, tumor growth of GR6 tumor model. *F*, mice bearing GR6 xenograft were treated with 35d (100 mg/kg), osi (2 mg/kg), or the combination (n = 3–5 per arm) as described in (*C*). *G*, a waterfall plot representation of the changes in tumor volume of each tumor taken at day 98 of the osi alone and the osi-35d combination treatment in the xenograft mice. Tumor volumes above 100% are truncated at 100%. Data in (*C*) and (*F*) represent mean ± SD. *p* values were determined by an unpaired, two-tailed Student’s *t* test (∗*p* < 0.05, ∗∗*p* < 0.01, ∗∗∗*p* < 0.001) (*D* and *G*). EGFR, epidermal growth factor receptor; gef, gefitinib; GR, gef-resistant; LUAD, lung adenocarcinoma; osi, osimertinib; TKI, tyrosine kinase inhibitor.

Our clinical analysis revealed that increased expression of HSP70 was positively correlated with improved OS in EGFR-mutant LUAD patients who received TKI treatment (Fig. 3*E*), suggesting that upregulation of hsp70 may enhance anticancer effects of TKIs. Moreover, we found that treatment with 35d effectively upregulated the expression of HSP70 in TKI-resistant cells (Figs. 3*D* and 4, *A*, *B*, and *H*), presenting a therapeutic opportunity for overcoming TKI resistance. Based on these findings, we hypothesized that treatment of 35d may reversed TKI resistance in EGFR-mutant LUADs. To investigate whether 35d can enhance anticancer activity of TKI in the TKI-resistant LUAD cells, we treated GR6 and GR10 cells with a combination of 35d and clinically used gef and osi (Fig. 6*A*). We assessed the effectiveness of the treatments, either alone or combination with 35d and TKI, by measuring cell killing activity using the 3-[4,5-dimethylthiazol2-yl]-2,5-diphenyltetrazolium bromide assay and calculating the combination index using CompuSyn (https://www.combosyn.com/) (33). Our results showed that treatment of 35d significantly enhanced anticancer activity of gef and osi in GR6 and GR10 cells (Fig. 6*A*). Importantly, we observed synergistic inhibitions of cell growth by combining 35d with gef and osi in these cells (Fig. 6*B*). Osi, a third generation TKI, has been proven as the first-line treatment and shown to prolong patient survival compared to first-generation TKIs in patients with untreated advanced EGFR-mutant NSCLC (34). To determine whether the combination therapy of 35d and TKI is effective in inhibiting acquired resistance to TKI *in vivo*, mice bearing TKI-sensitive HCC827 tumor were treated with 35d and osi orally. Treatment of osi (1 mg/kg) alone initially induced HCC827 tumor regression, but the treating tumors then regrew after 43 days of treatment, indicating the occurrence of *in vivo* tumor resistance to osi and recapitulating clinical osi resistance in the SCID mice models (Fig. 6*C*). The combination of 35d did not affect the initial tumor response to osi (day 1–day 43, Fig. 6*C*). Importantly, 35d-osi combination markedly inhibited tumor reprogression to osi after day 43 (Fig. 6, *C* and *D*) and significantly extended mice survival (Fig. 6*E*). These results suggested that 35d treatment effectively repressed the acquisition of TKI resistance and inhibited the resistant tumor growth.

To strengthen our findings, we evaluated the combination of 35d and osi in another xenograft mouse model bearing GR6 tumors, which had acquired resistance to first generation TKI gef *in vitro* (Fig. 1*J*) (12). While tumor growth of GR6 xenografts were initially suppressed by 2 mg/kg third generation TKI osi, our result showed that the treating tumors regrew rapidly after osi treatment for 57 days. However, the 35d-osi combination effectively limited tumor reprogression to osi (Fig. 6, *F* and *G*).

Furthermore, the effective doses of the 35d-osi combination did not significantly affect the values of the indicators for liver and kidney functions in mice after the treatments for 7 days (Fig. S2*A*). To examine the long-term effects of the osi-35d combination treatment, we monitored mice body weight during treatment period (Fig. S2*B*) and performed histological morphology analysis of multiple tissues from the mice after the treatments for more than 4 months (Fig. S2*C* and Table S5). The effective doses of the 35d-osi combination did not significantly affect mice body weight (Fig. S2*B*). The histological analysis by H&E staining showed skin and lung lesions in the mice treated with osi alone for 139 days (Table S5 and Fig. S2*C*), and the combination of 35d further minimize the osi-induced lesions in the mice (Table S5). All these data suggested that the 35d-osi combination at the doses administered is a safe and effective therapy for treating EGFR-mutant, TKI-resistant LUADs.

## Discussion

In clinical, LUAD tumors with WT EGFR rarely responded to TKIs (35). On the other hand, tumors with mutant EGFR usually sensitized to TKI as they rely heavily on EGFR kinase activity and downstream TKI-sensitive pathways for their growth (9). As a result, TKIs have been approved as the first-line treatment for EGFR-mutant LUAD patients. However, despite the initial response to EGFR kinase inhibitors, virtually all the EGFR-mutant patients eventually experience recurrence (9) due to the sustained TKI-insensitive EGFR signaling in the TKI-treated tumors, leading to recurrent tumor resistance (12). These clinical outcomes suggested that current EGFR therapies, which target EGFR kinase activity, are insufficient to cure LUADs. Identification of effective therapies for such resistant tumors is therefore an urgent medical need.

In addition to the genetic mutation status, upregulation of EGFR expression has been identified as a negative prognostic marker for the patient survival in NSCLC cancers (36, 37, 38, 39, 40, 41, 42, 43, 44). However, the correlations between EGFR expression and patient survival in specific subtypes of NSCLCs, such as LUAD and LUSC, remains inconclusive due to lacking of a comprehensive system for subtype comparison (45). In this study, we systematically investigated subtypes of NSCLC using TCGA and KM plotter datasets and found that higher EGFR expression levels were associated with shorter survival in LUAD patients, but not LUSC. Preclinical models have shown that EGFR, independent of its kinase activity, promotes tumor growth (12) and causes drug resistance through activation of TKI-insensitive EGFR signaling (12). These tumor cells rely on the EGFR protein–mediated, TKI-insensitive EGFR signaling, but not EGFR kinase activity, for survival and are insensitive to EGFR kinase inhibitors. However, they do sensitize to EGFR depletion, indicating that TKI-insensitive EGFR signaling is specifically presented in EGFR-mutant, TKI-resistant LUAD tumors. Our data supported the targeting of the TKI-insensitive EGFR signaling by a small molecule 35d as a potentially effective, safe, and feasible strategy for treating the LUAD tumors with multiple TKI resistance including EGFR C797S mutations.

Hsp70, a stress-inducible chaperone, plays a critical role in maintaining the functional proteins in normal tissues. However, its role in cancer remains a subject of debate (46). For example, hsp70 has been shown to promote gastric cancer cell growth by inhibiting apoptotic cell death (47). Downregulation of hsp70 by knockdown of heat shock transcription factor 1 (HSF-1) suppresses lung cancer cell growth (48). On the other hand, accumulation of hsp70 protein at lysosomal membranes has been shown to cause cancer cell death (49). Overexpression of hsp70 has been shown to maintain DNA repair activity in TKI-sensitive cells and delay acquisition of resistant mutation on *EGFR in vitro* (50). Here, higher hsp70 expression is correlated with longer survival in EGFR-mutant LUAD patients treated with TKI, suggesting a tumor-suppressive role of hsp70 in lung cancer patients. Importantly, 35d markedly induced hsp70 and selectively activated lysosome-dependent degradation pathway by upregulation of bag3 and p62, leading to reduced expression of oncoprotein EGFR.

Curcumin was reported to inhibit EGFR expression in *in vitro* study (23). As a single-agent therapy, curcumin has been shown to be a well-tolerated treatment, with doses of up to 6000 to 8000 mg/day for 2 months, and has ranched phase 3 clinical trials. Our study identified that a curcumin derivative 35d is more effective than curcumin at degrading EGFR protein and suppressing tumorigenesis in EGFR-mutant, TKI-resistant LUADs. Combining 35d with an EGFR kinase inhibitor could be an effective and safe treatment for TKI-resistant LUADs. Indeed, our preclinical models have shown that extended treatments of 35d (100 mg/kg/day) with osi for over 4 months are not only safe for mice but also effective in inhibiting tumor growth following tumor reprogression on osi.

While clinical tests are essential to determine the safety and dosage for 35d, our results will call for clinical trials testing the combination of 35d and clinical TKIs in EGFR-mutant LUAD patients with TKI resistance. A previous study demonstrated that a combination of TKI afatinib with cetuximab, which reduce EGFR protein expression, exhibited an encouraged tumor response in EGFR-mutant LUADs progressing on afatinib (51). Therefore, we propose that 35d-osi combination could be used in EGFR-mutant LUAD patients who have acquired resistance to prior-line TKIs or as a first-line therapy for naïve patients in the future.

## Experimental procedures

### Public datasets and analysis

For the TCGA LUAD and LUSC dataset, we obtained the RNA-seq, mutation, and copy number variation (CNV) data from DriverDBv3 (52). In sum, RNA-seq and exome sequencing data were downloaded from the Genomic Data Commons (GDC) data portal (https://portal.gdc.cancer.gov/). Level 3 CNV data were collected from the TCGA2BED tool (53). For the KM plotter LUAD and LUSC dataset, we used mRNA RNA-seq pan-cancer data and analyzed from KM plotter (http://kmplot.com/analysis/index.php?p=service&cancer=pancancer_rnaseq). For the East Asia cohort of LUAD, the mutation data and the clinical information were obtained from OncoSG (https://src.gisapps.org/OncoSG/). The R package, Survival (Version 2.41-3), was used to calculate the cox regression (or cox proportional hazards) model between two predefined groups. We stratified patients according to the medians of expression levels or the status of DNA CN. The significant criterion is log-rank *p* values <0.05.

### Tumorigenesis assays

GR6, GR8, VGHT 11, HCC827 cells were inoculated subcutaneously into the hind limbs of SCID mice. Tumor-bearing mice were randomized and were administered orally five times per week. Tumor volumes for subcutaneous tumors were measured with calipers twice per week as described previously (12). All animal procedures were conducted under the guidelines approved by the Institutional Animal Care and Use Committee at China Medical University.

### Cell culture

Human NSCLC cell lines (H460, H1299, H1650, HCC827, and H1975) were obtained from American Type Culture Collection (ATCC) and VGHT 11 cells were isolated from malignant pleural fluids of EGFR-mutant lung cancer patient as described previously (20), and the cells and their subclones were grown in RPMI medium supplemented with 10% fetal bovine serum (FBS). HCC827 GR cells were in standard RPMI medium in the presence of 1 μM gef. H3255 was a gift from Dr Zhen Fan and were grown in DMEM/F12 medium supplemented with 10% FBS. All cell lines have been tested for *mycoplasma* contamination and were validated by short tandem repeat DNA fingerprinting as described previously (12).

### Cell counting and cell viability assays

Cell counting or cell viability assays were used to estimate the cellular responses to the treatments, following previously established protocols (12). Briefly, for counting the cells, cells were seeded in 6-well plates in culture medium containing 10% FBS overnight, then treated with the respective agent(s) for indicated days. After treatment, the cells were harvested, diluted in 0.4% trypan blue solution, and counted using a hemocytometer. The total number of viable cells was calculated and recorded. For cell viability assay, cells were grown in flat-bottom 96-well plates with 100 μl of culture medium for each well in the presence or absence of compounds. After 24, 48, or 72 h of treatment, the cells were treated with 10 μl of 12 μM 3-[4,5-dimethylthiazol2-yl]-2,5-diphenyltetrazolium bromide and incubated for 2 to 4 h at 37 °C. Subsequently, 100 μl of solubilization solution (0.01 M HCl, 10% SDS) was added to each well and incubated overnight at 37 °C. The absorbance of the samples was determined using a spectrophotometer (at 550–600 nm). For the validated cell viability assays, cells were seeded in 24-well plates in RPMI 1640 medium containing 10% FBS overnight, then treated with the respective agent(s). After 6 days, the cells were washed with PBS, fixed with ice-cold methanol, and stained with 0.5% crystal violet. Crystal violet was dissolved in acetic acid and absorbance of each well measured at 570 nm (*A*_*570*_) using an ELISA plate reader. The average *A*_*570*_ of untreated cells was set to 100%. The percentage of treated cells that were viable was then calculated accordingly. The median IC_50_ for each drug was determined from the dose-effect relationship using the CompuSyn software (version 1.0.1; CompuSyn, Inc). The interactions of two drug treatments were evaluated by the Chou-Talalay combination indices (54).

### WB analysis

WB analysis was performed as described previously (12). Briefly, cells were washed twice with PBS, lysed in lysis buffer, proteins were then separated by SDS electrophoresis on a 10% or 12% polyacrylamide gel, and transferred onto polyvinylidene fluoride membranes (Invitrogen). After overnight incubation with primary antibody, washing, and incubation with secondary antibodies, blots were developed with a chemiluminescence system (Bio-Rad).

### Antibodies and reagents

The following were the antibodies used: anti-mouse tubulin (Genetex, GTX27291), anti-rabbit EGFR (abcam, ab52894), anti-rabbit pEGFR (abcam, ab5644), anti-rabbit hsp70 (Genetex, GTX104126), anti-rabbit bag3 (Genetex, GTX102396), and anti-rabbit p62 (Genetex, GTX100685). The following were the reagents used: ver-155008 (Medchem Express, HY-10941), pif (Medchem Express, HY-10940), bafilomycin A1 (Medchem Express, HY-100558), hydroxychloroquine (Medchem Express, HY-B1370), ixazomib (Medchem Express, HY-10453), MG132 (Medchem Express, HY-13259), osi (Medchem Express, HY-15772), and gef (LC lab, G-4408). The synthesis of all curcumin derivatives has been reported in our earlier publication (26). The structures and synthetic procedures of MTH-3, 21b, 22a, 22b, 23a, 24b, 25, 33, 35a, 35d, 36, and 37 correspond to the synthetic methods employed for the synthesis of compounds 9a, 10a, 9b, 10b, 11, 14a, 14b, 15, 16a, 16d, 17, and 18, respectively, as depicted in Scheme 1A-C of the reference (26). Briefly, curcumin derivatives were synthesized from curcumin *via* a three-step sequence involving esterification of curcumin with 2,2,5-trimethyl-1,3-dioxane-5-carboxylic acid to afford an ester intermediate, which was in turn dialkylated with alkyl bromide and hydrolysis with HCl(aq).

### RNA-seq analysis

The purified RNA from 35d-treated GR6 cells was used for the preparation of the sequencing library by TruSeq Stranded mRNA Library Prep Kit (Illumina) following the manufacturer’s recommendations. Briefly, mRNA was purified from total RNA (1 μg) by oligo(dT)-coupled magnetic beads and fragmented into small pieces under elevated temperature. The first-strand complementary DNA (cDNA) was synthesized using reverse transcriptase and random primers. After the generation of double-strand cDNA and adenylation on 3′ ends of DNA fragments, the adaptors were ligated and purified with AMPure XP system (Beckman Coulter). The quality of the libraries was assessed on the Agilent Bioanalyzer 2100 system and a real time PCR system. The qualified libraries were then sequenced on an Illumina NovaSeq 6000 platform with 150 bp paired-end reads generated by Genomics, BioSci & Tech Co. The bases with low quality and sequences from adapters in raw data were removed using program trimmomatic (http://www.usadellab.org/cms/?page=trimmomatic) (version 0.39) (55). The filtered reads were aligned to the reference genomes using Bowtie2 (version 2.3.4.1) (56). A user friendly software RSEM (https://deweylab.github.io/RSEM/) (version 1.2.28) was applied for the quantification of the transcript abundance (57). Differentially expressed genes were identified by EBSeq (version 1.16.0) (58).We utilized the overrepresentation method, as described in our previous studies, to identify the function of Gene Ontology (59) that enriched the significant genes. GSEA (60) was conducted by the R package, fgsea, which uses a fast algorithm to make more permutations to calculate the *p* value of the enrich score.

### Statistical analysis

All experiments were repeated at least three times unless stated otherwise. Error bars represent SD. Student’s *t* test was used to compare two groups of independent samples followed by the Bonferroni method to correct the likelihood of the observed data under the null hypothesis for multiple comparisons. The corrected *p* value is calculated by multiplying the original *p* value (obtained from a Student’s *t* test) by the number of comparisons made for each data analysis. Kaplan–Meier analysis with log-rank test was used to compare the survival of two groups. In all statistical analyses, a *p* value of less than 0.05 was considered significant.

## Data availability

All data are included in the article except the RNA-Seq data that was submitted to the Gene Expression Omnibus with the accession number GSE215786.

## Supporting information

This article contains supporting information (23, 61, 62, 63, 64, 65, 66, 67, 68, 69, 70, 71, 72, 73, 74, 75, 76, 77, 78, 79, 80, 81, 82, 83, 84, 85, 86, 87, 88, 89, 90, 91, 92, 93, 94, 95, 96, 97, 98, 99, 100).

## Conflict of interest

The authors declare that they have no conflicts of interest with the contents of this article.

## References

[1] Bade B.C., Dela Cruz C.S. (2020). Lung cancer 2020: epidemiology, etiology, and prevention. Clin. Chest Med..

[2] Shaurova T., Zhang L., Goodrich D.W., Hershberger P.A. (2020). Understanding lineage plasticity as a path to targeted therapy failure in EGFR-mutant non-small cell lung cancer. Front. Genet..

[3] Bartholomew C., Eastlake L., Dunn P., Yiannakis D. (2017). EGFR targeted therapy in lung cancer; an evolving story. Respir. Med. Case Rep..

[4] Cai W.Q., Zeng L.S., Wang L.F., Wang Y.Y., Cheng J.T., Zhang Y. (2020). The latest battles between EGFR monoclonal antibodies and resistant tumor cells. Front. Oncol..

[5] Hsu J.L., Hung M.C. (2016). The role of HER2, EGFR, and other receptor tyrosine kinases in breast cancer. Cancer Metastasis Rev..

[6] Chen C.H., Wang B.W., Hsiao Y.C., Wu C.Y., Cheng F.J., Hsia T.C. (2021). PKCdelta-mediated SGLT1 upregulation confers the acquired resistance of NSCLC to EGFR TKIs. Oncogene.

[7] Del Re M., Crucitta S., Gianfilippo G., Passaro A., Petrini I., Restante G. (2019). Understanding the mechanisms of resistance in EGFR-positive NSCLC: from tissue to liquid biopsy to guide treatment strategy. Int. J. Mol. Sci..

[8] Chen M.K., Hung M.C. (2016). Regulation of therapeutic resistance in cancers by receptor tyrosine kinases. Am. J. Cancer Res..

[9] Rotow J., Bivona T.G. (2017). Understanding and targeting resistance mechanisms in NSCLC. Nat. Rev. Cancer.

[10] Minari R., Bordi P., Tiseo M. (2016). Third-generation epidermal growth factor receptor-tyrosine kinase inhibitors in T790M-positive non-small cell lung cancer: review on emerged mechanisms of resistance. Transl. Lung Cancer Res..

[11] Piotrowska Z., Niederst M.J., Karlovich C.A., Wakelee H.A., Neal J.W., Mino-Kenudson M. (2015). Heterogeneity underlies the emergence of EGFRT790 wild-type clones following treatment of T790M-positive cancers with a third-generation EGFR inhibitor. Cancer Discov..

[12] Lee P.C., Fang Y.F., Yamaguchi H., Wang W.J., Chen T.C., Hong X. (2018). Targeting PKCdelta as a therapeutic strategy against heterogeneous mechanisms of EGFR inhibitor resistance in EGFR-mutant lung cancer. Cancer Cell.

[13] Weihua Z., Tsan R., Huang W.C., Wu Q., Chiu C.H., Fidler I.J. (2008). Survival of cancer cells is maintained by EGFR independent of its kinase activity. Cancer Cell.

[14] To C., Jang J., Chen T., Park E., Mushajiang M., De Clercq D.J.H. (2019). Single and dual targeting of mutant EGFR with an allosteric inhibitor. Cancer Discov..

[15] Lynch T.J., Bell D.W., Sordella R., Gurubhagavatula S., Okimoto R.A., Brannigan B.W. (2004). Activating mutations in the epidermal growth factor receptor underlying responsiveness of non-small-cell lung cancer to gefitinib. N. Engl. J. Med..

[16] Shepherd F.A., Tsao M.S. (2006). Unraveling the mystery of prognostic and predictive factors in epidermal growth factor receptor therapy. J. Clin. Oncol..

[17] Tomasello C., Baldessari C., Napolitano M., Orsi G., Grizzi G., Bertolini F. (2018). Resistance to EGFR inhibitors in non-small cell lung cancer: clinical management and future perspectives. Crit. Rev. Oncol. Hematol..

[18] Nagy A., Munkacsy G., Gyorffy B. (2021). Pancancer survival analysis of cancer hallmark genes. Sci. Rep..

[19] Yang Z., Yang N., Ou Q., Xiang Y., Jiang T., Wu X. (2018). Investigating novel resistance mechanisms to third-generation EGFR tyrosine kinase inhibitor osimertinib in non-small cell lung cancer patients. Clin. Cancer Res..

[20] Lin C.Y., Huang K.Y., Lin Y.C., Yang S.C., Chung W.C., Chang Y.L. (2021). Vorinostat combined with brigatinib overcomes acquired resistance in EGFR-C797S-mutated lung cancer. Cancer Lett..

[21] Bivona T.G., Hieronymus H., Parker J., Chang K., Taron M., Rosell R. (2011). FAS and NF-kappaB signalling modulate dependence of lung cancers on mutant EGFR. Nature.

[22] Sos M.L., Koker M., Weir B.A., Heynck S., Rabinovsky R., Zander T. (2009). PTEN loss contributes to erlotinib resistance in EGFR-mutant lung cancer by activation of Akt and EGFR. Cancer Res..

[23] Lee J.Y., Lee Y.M., Chang G.C., Yu S.L., Hsieh W.Y., Chen J.J. (2011). Curcumin induces EGFR degradation in lung adenocarcinoma and modulates p38 activation in intestine: the versatile adjuvant for gefitinib therapy. PLoS One.

[24] Chen P., Huang H.P., Wang Y., Jin J., Long W.G., Chen K. (2019). Curcumin overcome primary gefitinib resistance in non-small-cell lung cancer cells through inducing autophagy-related cell death. J. Exp. Clin. Cancer Res..

[25] Liu W., Zhai Y., Heng X., Che F.Y., Chen W., Sun D. (2016). Oral bioavailability of curcumin: problems and advancements. J. Drug Target.

[26] Hsieh M.T., Chang L.C., Hung H.Y., Lin H.Y., Shih M.H., Tsai C.H. (2017). New bis(hydroxymethyl) alkanoate curcuminoid derivatives exhibit activity against triple-negative breast cancer *in vitro* and *in vivo*. Eur. J. Med. Chem..

[27] Chang L.C., Hsieh M.T., Yang J.S., Lu C.C., Tsai F.J., Tsao J.W. (2018). Effect of bis(hydroxymethyl) alkanoate curcuminoid derivative MTH-3 on cell cycle arrest, apoptotic and autophagic pathway in triple-negative breast adenocarcinoma MDA-MB-231 cells: an *in vitro* study. Int. J. Oncol..

[28] Chen J., Yang H., Teo A.S.M., Amer L.B., Sherbaf F.G., Tan C.Q. (2020). Genomic landscape of lung adenocarcinoma in East Asians. Nat. Genet..

[29] Seo J., Han S.Y., Seong D., Han H.J., Song J. (2019). Multifaceted C-terminus of HSP70-interacting protein regulates tumorigenesis via protein quality control. Arch. Pharm. Res..

[30] Chung C., Yoo G., Kim T., Lee D., Lee C.S., Cha H.R. (2016). The E3 ubiquitin ligase CHIP selectively regulates mutant epidermal growth factor receptor by ubiquitination and degradation. Biochem. Biophys. Res. Commun..

[31] Minoia M., Boncoraglio A., Vinet J., Morelli F.F., Brunsting J.F., Poletti A. (2014). BAG3 induces the sequestration of proteasomal clients into cytoplasmic puncta: implications for a proteasome-to-autophagy switch. Autophagy.

[32] Kettern N., Rogon C., Limmer A., Schild H., Hohfeld J. (2011). The Hsc/Hsp70 co-chaperone network controls antigen aggregation and presentation during maturation of professional antigen presenting cells. PLoS One.

[33] Lee P.C., Lee H.J., Kakadiya R., Sanjiv K., Su T.L., Lee T.C. (2013). Multidrug-resistant cells overexpressing P-glycoprotein are susceptible to DNA crosslinking agents due to attenuated Src/nuclear EGFR cascade-activated DNA repair activity. Oncogene.

[34] Ramalingam S.S., Vansteenkiste J., Planchard D., Cho B.C., Gray J.E., Ohe Y. (2020). Overall survival with osimertinib in untreated, EGFR-mutated advanced NSCLC. N. Engl. J. Med..

[35] Westover D., Zugazagoitia J., Cho B.C., Lovly C.M., Paz-Ares L. (2018). Mechanisms of acquired resistance to first- and second-generation EGFR tyrosine kinase inhibitors. Ann. Oncol..

[36] O'Farrell H., Harbourne B., Kurlawala Z., Inoue Y., Nagelberg A.L., Martinez V.D. (2019). Integrative genomic analyses identifies GGA2 as a cooperative driver of EGFR-mediated lung tumorigenesis. J. Thorac. Oncol..

[37] Okabe T., Okamoto I., Tamura K., Terashima M., Yoshida T., Satoh T. (2007). Differential constitutive activation of the epidermal growth factor receptor in non-small cell lung cancer cells bearing EGFR gene mutation and amplification. Cancer Res..

[38] Liang Z., Zhang J., Zeng X., Gao J., Wu S., Liu T. (2010). Relationship between EGFR expression, copy number and mutation in lung adenocarcinomas. BMC Cancer.

[39] Chang J.W., Liu H.P., Hsieh M.H., Fang Y.F., Hsieh M.S., Hsieh J.J. (2008). Increased epidermal growth factor receptor (EGFR) gene copy number is strongly associated with EGFR mutations and adenocarcinoma in non-small cell lung cancers: a chromogenic *in situ* hybridization study of 182 patients. Lung Cancer.

[40] Shan L., Wang Z., Guo L., Sun H., Qiu T., Ling Y. (2015). Concurrence of EGFR amplification and sensitizing mutations indicate a better survival benefit from EGFR-TKI therapy in lung adenocarcinoma patients. Lung Cancer.

[41] Zhang X., Zhang Y., Tang H., He J. (2017). EGFR gene copy number as a predictive/biomarker for patients with non-small-cell lung cancer receiving tyrosine kinase inhibitor treatment: a systematic review and meta-analysis. J. Investig. Med..

[42] Hirsch F.R., Varella-Garcia M., Bunn P.A., Di Maria M.V., Veve R., Bremmes R.M. (2003). Epidermal growth factor receptor in non-small-cell lung carcinomas: correlation between gene copy number and protein expression and impact on prognosis. J. Clin. Oncol..

[43] Dacic S., Flanagan M., Cieply K., Ramalingam S., Luketich J., Belani C. (2006). Significance of EGFR protein expression and gene amplification in non–small cell lung carcinoma. Am. J. Clin. Pathol..

[44] Jeon Y.K., Sung S.W., Chung J.H., Park W.S., Seo J.W., Kim C.W. (2006). Clinicopathologic features and prognostic implications of epidermal growth factor receptor (EGFR) gene copy number and protein expression in non-small cell lung cancer. Lung Cancer.

[45] Meert A.P., Martin B., Delmotte P., Berghmans T., Lafitte J.J., Mascaux C. (2002). The role of EGF-R expression on patient survival in lung cancer: a systematic review with meta-analysis. Eur. Respir. J..

[46] Vostakolaei M.A., Hatami-Baroogh L., Babaei G., Molavi O., Kordi S., Abdolalizadeh J. (2021). Hsp70 in cancer: a double agent in the battle between survival and death. J. Cell Physiol..

[47] Xiang T.X., Li Y., Jiang Z., Huang A.L., Luo C., Zhan B. (2008). RNA interference-mediated silencing of the Hsp70 gene inhibits human gastric cancer cell growth and induces apoptosis *in vitro* and *in vivo*. Tumori.

[48] Lee S., Jung J., Lee Y.J., Kim S.K., Kim J.A., Kim B.K. (2021). Targeting HSF1 as a therapeutic strategy for multiple mechanisms of EGFR inhibitor resistance in EGFR mutant non-small-cell lung cancer. Cancers (Basel).

[49] Doulias P.T., Kotoglou P., Tenopoulou M., Keramisanou D., Tzavaras T., Brunk U. (2007). Involvement of heat shock protein-70 in the mechanism of hydrogen peroxide-induced DNA damage: the role of lysosomes and iron. Free Radic. Biol. Med..

[50] Cao X., Zhou Y., Sun H., Xu M., Bi X., Zhao Z. (2018). EGFR-TKI-induced HSP70 degradation and BER suppression facilitate the occurrence of the EGFR T790M resistant mutation in lung cancer cells. Cancer Lett..

[51] Janjigian Y.Y., Smit E.F., Groen H.J., Horn L., Gettinger S., Camidge D.R. (2014). Dual inhibition of EGFR with afatinib and cetuximab in kinase inhibitor-resistant EGFR-mutant lung cancer with and without T790M mutations. Cancer Discov..

[52] Liu S.H., Shen P.C., Chen C.Y., Hsu A.N., Cho Y.C., Lai Y.L. (2020). DriverDBv3: a multi-omics database for cancer driver gene research. Nucleic Acids Res..

[53] Cumbo F., Fiscon G., Ceri S., Masseroli M., Weitschek E. (2017). TCGA2BED: extracting, extending, integrating, and querying The Cancer Genome Atlas. BMC Bioinformatics.

[54] Chou T.C. (2006). Theoretical basis, experimental design, and computerized simulation of synergism and antagonism in drug combination studies. Pharmacol. Rev..

[55] Bolger A.M., Lohse M., Usadel B. (2014). Trimmomatic: a flexible trimmer for Illumina sequence data. Bioinformatics.

[56] Langmead B., Salzberg S.L. (2012). Fast gapped-read alignment with Bowtie 2. Nat. Methods.

[57] Li B., Dewey C.N. (2011). RSEM: accurate transcript quantification from RNA-seq data with or without a reference genome. BMC Bioinformatics.

[58] Leng N., Dawson J.A., Thomson J.A., Ruotti V., Rissman A.I., Smits B.M. (2013). EBSeq: an empirical Bayes hierarchical model for inference in RNA-seq experiments. Bioinformatics.

[59] The Gene Ontology Consortium (2019). The Gene Ontology Resource: 20 years and still GOing strong. Nucleic Acids Res..

[60] Subramanian A., Tamayo P., Mootha V.K., Mukherjee S., Ebert B.L., Gillette M.A. (2005). Gene set enrichment analysis: a knowledge-based approach for interpreting genome-wide expression profiles. Proc. Natl. Acad. Sci. U. S. A..

[61] Lev-Ari S., Starr A., Vexler A., Karaush V., Loew V., Greif J. (2006). Inhibition of pancreatic and lung adenocarcinoma cell survival by curcumin is associated with increased apoptosis, down-regulation of COX-2 and EGFR and inhibition of Erk1/2 activity. Anticancer Res.

[62] Wada K., Lee J.-Y., Hung H.-Y., Shi Q., Lin L., Zhao Y. (2015). Novel curcumin analogs to overcome EGFR–TKI lung adenocarcinoma drug resistance and reduce EGFR–TKI-induced GI adverse effects. Bioorg Med Chem.

[63] Chadalapaka G., Jutooru I., Burghardt R., Safe S. (2010). Drugs that target specificity proteins downregulate epidermal growth factor receptor in bladder cancer cells. Mol Cancer Res.

[64] Zhang L., Tao X., Fu Q., Ge C., Li R., Li Z. (2019). Curcumin inhibits cell proliferation and migration in NSCLC through a synergistic effect on the TLR4/MyD88 and EGFR pathways. Oncol Rep.

[65] Cai Y., Sheng Z., Liang S. (2019). Radiosensitization effects of curcumin plus cisplatin on non-small cell lung cancer A549 cells. Oncol Lett.

[66] SomersEdgar T.J., Scandlyn M.J., Stuart E.C., Le Nedelec M.J., Valentine S.P., Rosengren R.J. (2008). The combination of epigallocatechin gallate and curcumin suppresses ER alpha-breast cancer cell growth in vitro and in vivo. Int J Cancer.

[67] Giommarelli C., Zuco V., Favini E., Pisano C., Dal Piaz F., De Tommasi N. (2010). The enhancement of antiproliferative and proapoptotic activity of HDAC inhibitors by curcumin is mediated by Hsp90 inhibition. Cell Mol Life Sci.

[68] Gandhy S.U., Kim K., Larsen L., Rosengren R.J., Safe S. (2012). Curcumin and synthetic analogs induce reactive oxygen species and decreases specificity protein (Sp) transcription factors by targeting microRNAs. BMC Cancer.

[69] Jiang X., Huang Y. (2020). Curcumin derivative C086 combined with cisplatin inhibits proliferation of osteosarcoma cells. Med Sci Monit.

[70] Yang J., Zhu D., Liu S., Shao M., Liu Y., Li A. (2020). Curcumin enhances radiosensitization of nasopharyngeal carcinoma by regulating circRNA network. Mol Carcinog.

[71] Zhou J., Zhao T., Ma L., Liang M., Guo Y.J., Zhao L.M. (2017). Cucurbitacin B and SCH772984 exhibit synergistic anti-pancreatic cancer activities by suppressing EGFR, PI3K/Akt/mTOR, STAT3 and ERK signaling. Oncotarget.

[72] Klungsaeng S., Kukongviriyapan V., Prawan A., Kongpetch S., Senggunprai L. (2020). Targeted modulation of FAK/PI3K/PDK1/AKT and FAK/p53 pathways by cucurbitacin B for the antiproliferation effect against human cholangiocarcinoma cells. Am J Chin Med.

[73] Gupta P., Srivastava S.K. (2014). Inhibition of Integrin-HER2 signaling by Cucurbitacin B leads to in vitro and in vivo breast tumor growth suppression. Oncotarget.

[74] Baba Y., Fujii M., Maeda T., Suzuki A., Yuzawa S., Kato Y. (2015). Deguelin induces apoptosis by targeting both EGFR-Akt and IGF1RAkt pathways in head and neck squamous cell cancer cell lines. BioMed Research International.

[75] Mehta R., Katta H., Alimirah F., Patel R., Murillo G., Peng X. (2013). Deguelin action involves c-Met and EGFR signaling pathways in triple negative breast cancer cells. PLoS One.

[76] Farabegoli F., Govoni M., Spisni E., Papi A. (2017). EGFR inhibition by (-)-epigallocatechin-3-gallate and IIF treatments reduces breast cancer cell invasion. Biosci Rep.

[77] Hou Z., Sang S., You H., Lee M.-J., Hong J., Chin K.-V. (2005). Mechanism of action of (−)-epigallocatechin-3-gallate: auto-oxidation–dependent inactivation of epidermal growth factor receptor and direct effects on growth inhibition in human esophageal cancer KYSE 150 cells. Cancer Res.

[78] Ma Y.C., Li C., Gao F., Xu Y., Jiang Z.B., Liu J.X. (2014). Epigallocatechin gallate inhibits the growth of human lung cancer by directly targeting the EGFR signaling pathway. Oncol Rep.

[79] Weng L.X., Wang G.H., Yao H., Yu M.F., Lin J. (2017). Epigallocatechin gallate inhibits the growth of salivary adenoid cystic carcinoma cells via the EGFR/Erk signal transduction pathway and the mitochondria apoptosis pathway. Neoplasma.

[80] Zhu W., Li M.C., Wang F.R., Mackenzie G.G., Oteiza P.I. (2020). The inhibitory effect of ECG and EGCG dimeric procyanidins on colorectal cancer cells growth is associated with their actions at lipid rafts and the inhibition of the epidermal growth factor receptor signaling. Biochem Pharmacol.

[81] Cromie M.M., Liu Z., Gao W. (2017). Epigallocatechin-3- gallate augments the therapeutic effects of benzo[a]pyrene-mediated lung carcinogenesis. Biofactors.

[82] Singh T., Gupta N.A., Xu S., Prasad R., Velu S.E., Katiyar S.K. (2015). Honokiol inhibits the growth of head and neck squamous cell carcinoma by targeting epidermal growth factor receptor. Oncotarget.

[83] Leeman-Neill R.J., Cai Q., Joyce S.C., Thomas S.M., Bhola N.E., Neill D.B. (2010). Honokiol inhibits epidermal growth factor receptor signaling and enhances the antitumor effects of epidermal growth factor receptor inhibitors. Clin Cancer Res.

[84] Wang X., Beitler J.J., Wang H., Lee M.J., Huang W., Koenig L. (2014). Honokiol enhances paclitaxel efficacy in multi-drug resistant human cancer model through the induction of apoptosis. PLoS One.

[85] Song J.M., Anandharaj A., Upadhyaya P., Kirtane A.R., Kim J.H., Hong K.H. (2016). Honokiol suppresses lung tumorigenesis by targeting EGFR and its downstream effectors. Oncotarget.

[86] Fan Y., Xue W., Schachner M., Zhao W. (2018). Honokiol eliminates glioma/glioblastoma stem cell-like cells via JAK-STAT3 signaling and inhibits tumor progression by targeting epidermal growth factor receptor. Cancers (Basel).

[87] Yang J., Pei H., Luo H., Fu A., Yang H., Hu J. (2017). Non-toxic dose of liposomal honokiol suppresses metastasis of hepatocellular carcinoma through destabilizing EGFR and inhibiting the downstream pathways. Oncotarget.

[88] Yang J., Wu W., Wen J., Ye H., Luo H., Bai P. (2017). Liposomal honokiol induced lysosomal degradation of Hsp90 client proteins and protective autophagy in both gefitinib-sensitive and gefitinib-resistant NSCLC cells. Biomaterials.

[89] Dai X., Li R.Z., Jiang Z.B., Wei C.L., Luo L.X., Yao X.J. (2018). Honokiol inhibits proliferation, invasion and induces apoptosis through targeting lyn kinase in human lung adenocarcinoma cells. Front Pharmacol.

[90] Okuda K., Umemura A., Umemura S., Kataoka S., Taketani H., Seko Y. (2021). Honokiol prevents non-alcoholic steatohepatitis-induced liver cancer via EGFR degradation through the glucocorticoid receptor-MIG6 axis. Cancers (Basel).

[91] Gomathinayagam R., Sowmyalakshmi S., Mardhatillah F., Kumar R., Akbarsha M.A., Damodaran C. (2008). Anticancer mechanism of plumbagin, a natural compound, on non-small cell lung cancer cells. Anticancer Res.

[92] Hafeez B.B., Jamal M.S., Fischer J.W., Mustafa A., Verma A.K. (2012). Plumbagin, a plant derived natural agent inhibits the growth of pancreatic cancer cells in in vitro and in vivo via targeting EGFR, Stat3 and NF-kappaB signaling pathways. Int J Cancer.

[93] Zhang G.H., Cai L.J., Wang Y.F., Zhou Y.H., An Y.F., Liu Y.C. (2013). Novel compound PS-101 exhibits selective inhibition in non-small-cell lung cancer cell by blocking the EGFR-driven antiapoptotic pathway. Biochem Pharmacol.

[94] Jung J.H., Lee J.O., Kim J.H., Lee S.K., You G.Y., Park S.H. (2010). Quercetin suppresses HeLa cell viability via AMPK-induced HSP70 and EGFR down-regulation. J Cell Physiol.

[95] Bhat F.A., Sharmila G., Balakrishnan S., Arunkumar R., Elumalai P., Suganya S., Raja Singh P., Srinivasan N., Arunakaran J. (2014). Quercetin reverses EGF-induced epithelial to mesenchymal transition and invasiveness in prostate cancer (PC-3) cell line via EGFR/PI3K/Akt pathway. J Nutr Biochem.

[96] Balakrishnan S., Mukherjee S., Das S., Bhat F.A., Raja Singh P., Patra C.R. (2017). Gold nanoparticles-conjugated quercetin induces apoptosis via inhibition of EGFR/PI3K/Akt-mediated pathway in breast cancer cell lines (MCF-7 and MDA-MB-231). Cell Biochem Funct.

[97] Wang G., Dai F., Yu K., Jia Z., Zhang A., Huang Q. (2015). Resveratrol inhibits glioma cell growth via targeting oncogenic microRNAs and multiple signaling pathways. Int J Oncol.

[98] Jin Z., Feng W., Ji Y., Jin L. (2017). Resveratrol mediates cell cycle arrest and cell death in human esophageal squamous cell carcinoma by directly targeting the EGFR signaling pathway. Oncol Lett.

[99] Chen C.Y., Yu Z.Y., Chuang Y.S., Huang R.M., Wang T.C. (2015). Sulforaphane attenuates EGFR signaling in NSCLC cells. J Biomed Sci.

[100] Pledgie-Tracy A., Sobolewski M.D., Davidson N.E. (2007). Sulforaphane induces cell type-specific apoptosis in human breast cancer cell lines. Mol Cancer Ther.

