# Supplementary material for: Diarylheptanoid 35d overcomes EGFR TKI resistance by inducing hsp70-mediated lysosomal degradation of EGFR in EGFR-mutant lung adenocarcinoma
Source: J Biol Chem. 2023 May 11;299(6):104814. doi: 10.1016/j.jbc.2023.104814 (PMC10276293; doi:10.1016/j.jbc.2023.104814)
Supplement: Supporting information [file mmc1.docx]

**Supporting information**

**Diarylheptanoid 35d overcomes EGFR TKI resistance by inducing hsp70-mediated lysosomal degradation of EGFR in EGFR-mutant lung adenocarcinoma**

**Table S1. Related to Table 1.**

Emerged natural compounds that reduce EGFR expression in cancer cells.

| **Compound** | **Ref.** |
| --- | --- |
| Curcumin and its derivatives | (23,61-71) |
| Cucurbitacin B | (72-74) |
| Deguelin | (75,76) |
| (-)-Epigallocatechin-3-gallate | (66,77-82) |
| Honokiol/  liposomal honokiol | (83-91) |
| Plumbagin and  its derivative | (92-94) |
| Quercetin /  gold nanoparticles-conjugated quercetin | (95-97) |
| Resveratrol | (98,99) |
| Sulforaphane | (100,101) |

The compounds in this table were showed to reduce EGFR expression in more than one publication.

**Table S2.** **Related to Figure 2A.**

Dose ranges of the compound candidates listed in Table 1 and tested in clinical trials

|  | **Numbers of clinical trials in ClinicalTrials.gov** | | | | | **Dose ranges (mg/day)** |
| --- | --- | --- | --- | --- | --- | --- |
| **Compound** | **Total** | **Trials in cancers** | **Trials in NSCLC** | **Phase 3 trials** | **Phase 3 trials in cancers** |  |
| Curcumin and its derivatives | 273 | 33 | 1 | 28 | 4 | 80–10,000 |
| Resveratrol | 186 | 8 | – | 17 | – | 20–5,000 |
| (-)-Epigallocatechin-3-gallate and tea extract | 118 | 16 | – | 15 | 1 | 200–1200 |
| Quercetin | 99 | 5 | – | 5 | – | 120–4,000 |
| Sulforaphane and broccoli seed extract | 82 | 10 | – | 2 | – | 0.05–0.2 |

**Table S3. Related to Figure 3.**

List of the identified genes in the top 10 gene sets analyzed from RNA-seq data.

| **Top 10 gene sets** | **Genes** |
| --- | --- |
| GO:0009408  Response to heat | HMOX1, HSPA1A, HSPA1B, HSPA6, PTGS2, THBS1, TRPV3, HSPB8, HSP90AA1, BAG3, HSPA8, HSPH1, CDKN1A, CCL2, GCLC, IL6, DNAJB1, BAG1, MICA |
| GO:0006986  Response to unfolded protein | HSPA1A, HSPA6, ATF3, CTH, THBS1, PPP1R15A, ASNS, HSP90AA1, DDIT3, HSPA8, HSPH1, IGFBP1, CCL2, GFPT1, CREB3L1, CHAC1, TSPYL2, DNAJB1, TMEM129 |
| GO:0035966  Response to topologically incorrect protein | HSPA1A, HSPA6, ATF3, CTH, THBS1, PPP1R15A, ASNS, HSP90AA1, DDIT3, HSPA8, HSPH1, IGFBP1, CCL2, GFPT1, CREB3L1, CHAC1, TSPYL2, DNAJB1, TMEM129, HDAC6 |
| GO:0048514  Blood vessel morphogenesis | ANGPTL4, EREG, HMOX1, CYP1B1, PTGS2, SERPINE1, THBS2, THBS1, HPGD, BMP4, BMPER, ADGRB2, NPPB, MYLK, PLXDC1, CCL2, ANPEP, ADM2, WNT7B, LAMA5, IL1B, EGF, IL6, NRP1, TGFB2, HSPG2, ZMIZ1, SAT1, CTGF, EDN1, COL3A1, TNFSF12, HOXA3 |
| GO:0034605  Cellular response to heat | HMOX1, HSPA1A, HSPA1B, HSPA6, PTGS2, THBS1, HSPB8, HSP90AA1, BAG3, HSPA8, HSPH1, CDKN1A, DNAJB1, BAG1 |
| GO:0042026  Protein refolding | HSPA1A, HSPA1B, HSPA6, HSP90AA1, HSPA8, DNAJB1, BAG1 |
| GO:0001525  Angiogenesis | ANGPTL4, EREG, HMOX1, CYP1B1, PTGS2, SERPINE1, THBS2, THBS1, BMP4, BMPER, ADGRB2, NPPB, PLXDC1, CCL2, ANPEP, ADM2, LAMA5, IL1B, EGF, IL6, NRP1, TGFB2, HSPG2, SAT1, CTGF, EDN1, TNFSF12, HOXA3 |
| GO:2001236  Regulation of extrinsic apoptotic signaling pathway | GCLM, HMOX1, HSPA1A, HSPA1B, SERPINE1, ATF3, THBS1, DAPK1, BMP4, IL1B, GCLC, NRP1, TGFB2, HYAL2, ARHGEF2, TNFSF12 |
| GO:0097191  Extrinsic apoptotic signaling pathway | GCLM, HMOX1, HSPA1A, HSPA1B, SERPINE1, ATF3, THBS1, MLLT11, DAPK1, BMP4, BAG3, IL1B, ERBB3, GCLC, NRP1, TGFB2, HYAL2, ARHGEF2, TNFSF12 |
| GO:2001233  Regulation of apoptotic signaling pathway | GCLM, HMOX1, HSPA1A, HSPA1B, PTGS2, SERPINE1, ATF3, CTH, THBS1, MLLT11, DAPK1, BMP4, DDIT3, S100A9, HSPH1, MUC1, INHBB, IL1B, CREB3L1, ANKRD2, GCLC, NRP1, TGFB2, HYAL2, ARHGEF2, TNFSF12 |

**Table S4. Related to Figure 3E.**

Correlation analysis of 15 HSP70 family gene expressions with LUAD patient overall survival.

| **EGFR status:** | Activating-mutant | | Wide-type |
| --- | --- | --- | --- |
| **TKI treatment:** | Yes (n=34) | No (n=57) | No (n=78) |
|  | **P-values** | | |
| HSPA1A | 0.611 | 0.377 | 0.826 |
| HSPA1B | 0.011 | 0.998 | 0.964 |
| HSPA1L | 0.479 | 0.061 | 0.225 |
| HSPA2 | 0.595 | 0.937 | 0.055 |
| HSPA4 | NA | 0.414 | 0.185 |
| HSPA4L | 0.434 | 0.947 | 0.799 |
| HSPA5 | 0.964 | 0.235 | 0.223 |
| HSPA6 | 0.786 | 0.408 | 0.581 |
| HSPA7 | NA | NA | NA |
| HSPA8 | 0.561 | 0.787 | 0.698 |
| HSPA9 | 0.071 | 0.949 | 0.067 |
| HSPA12A | 0.591 | 0.006 | 0.496 |
| HSPA12B | 0.171 | 0.516 | 0.721 |
| HSPA13 | 0.317 | 0.299 | 0.793 |
| HSPA14 | 0.668 | 0.433 | 0.024 |

NA, not available.

**
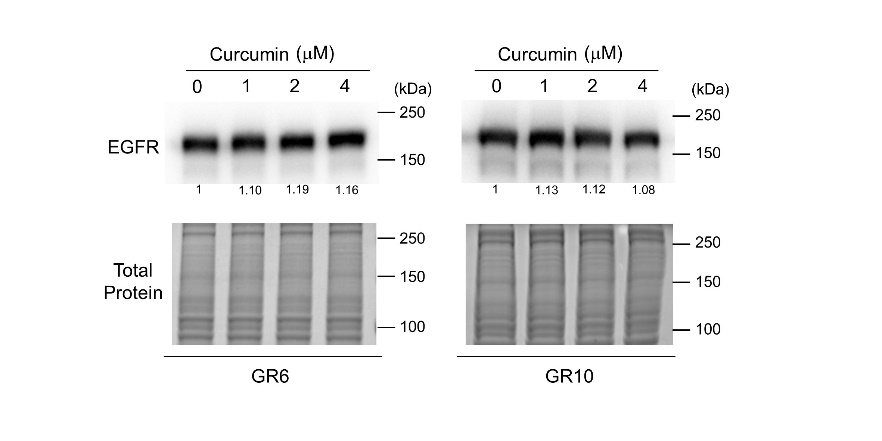
**

**Figure S1. Related to Fig. 4.** EGFR expression in GR6 and GR10 cells treated with curcumin for 24 h were determined by western blot analysis. Relative protein levels are indicated below the blot. Total protein was used as the loading control. Molecular weight markers are noted next to all immunoblots.

**
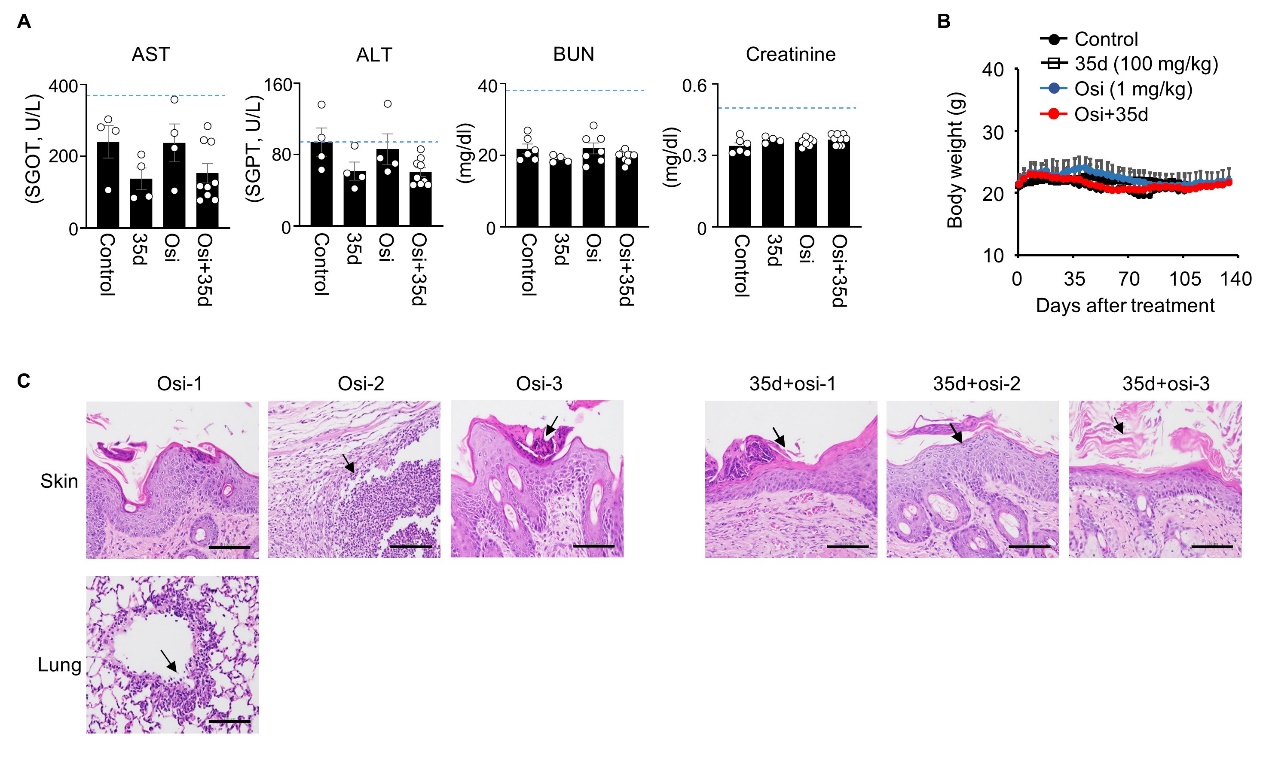
**

**Figure S2. Related to Figure 6.** *A*, Indicators for liver and kidney functions in each treatment group after drug treatment for 1 weeks. The normal range of aspartate aminotransaminase (AST), alanine aminotransaminase (ALT), blood urea nitrogen (BUN), and creatinine are 63–253 U/L, 35–90 U/L, 17–38 mg/dl, and 0.3–0.5 mg/dl, respectively. Data represent mean ± SD (n = 4-9). *B*, Body weight changes. Data represent mean ± SD (n = 7-9 per arm). *C*, Representative microscopy images (400x) of histopathologic lesions in skin and lung. H&E staining analysis in SCID mice treated with osi (1 mg/kg) alone and combination of osi and 35d (100 mg/kg) at day 139. The scale bar represents 100 μm.

**Table S5. Related to Figure 6.**

Histopathological analysis in the mice treated with osi alone and osi-35d combination for 139 days.

| **Organ** | **Histopathological findings** | **Grades** | |
| --- | --- | --- | --- |
|  |  | **Osi** | **Osi+35d** |
| Skin | Abscess | 0/5/0 | 0/0/0 |
|  | Acanthosis | 3/2/3 | 1/2/2 |
|  | Crust | 1/2/1 | 1/1/1 |
|  | Hyperkeratosis | 1/1/1 | 1/1/2 |
|  | Inflammation | 2/1/2 | 1/1/1 |
| Esophagus |  | 0/0/0 | 0/0/0 |
| Trachea |  | 0/0/0 | 0/0/0 |
| Lung | Perivascular cuffing | 2/0/0 | 0/0/0 |
| Stomach |  | 0/0/0 | 0/0/0 |
| Small intestine: |  |  |  |
| Duodenum |  | 0/0/0 | 0/0/0 |
| Ileum |  | 0/0/0 | 0/0/0 |
| Jejunum |  | 0/0/0 | 0/0/0 |
| Large intestine: |  |  |  |
| Cecum |  | 0/0/0 | 0/0/0 |
| Colon |  | 0/0/0 | 0/0/0 |
| Rectum |  | 0/0/0 | 0/0/0 |

3 mice per group after indicated treatments for 139 days were subjected to mouse histopathologic analysis.

Degree of lesions was graded from 0 to 5 depending on severity:

0 = no effect;

1 = minimal (< 1%);

2 = slight (1-25%);

3 = moderate (26-50%);

4 = moderate/severe (51-75%);

5 = severe/high (76-100%).

**Supplement Reference**

61. Lev-Ari, S., Starr, A., Vexler, A., Karaush, V., Loew, V., Greif, J., Fenig, E., Aderka, D., and Ben-Yosef, R. (2006) Inhibition of pancreatic and lung adenocarcinoma cell survival by curcumin is associated with increased apoptosis, down-regulation of COX-2 and EGFR and inhibition of Erk1/2 activity. *Anticancer Res* **26**, 4423-4430

62. Wada, K., Lee, J.-Y., Hung, H.-Y., Shi, Q., Lin, L., Zhao, Y., Goto, M., Yang, P.-C., Kuo, S.-C., Chen, H.-W., and Lee, K.-H. (2015) Novel curcumin analogs to overcome EGFR–TKI lung adenocarcinoma drug resistance and reduce EGFR–TKI-induced GI adverse effects. *Bioorganic & Medicinal Chemistry* **23**, 1507-1514

63. Chadalapaka, G., Jutooru, I., Burghardt, R., and Safe, S. (2010) Drugs that target specificity proteins downregulate epidermal growth factor receptor in bladder cancer cells. *Mol Cancer Res* **8**, 739-750

64. Zhang, L., Tao, X., Fu, Q., Ge, C., Li, R., Li, Z., Zhu, Y., Tian, H., Li, Q., Liu, M., Hu, H., Zeng, B., Lin, Z., Li, C., Luo, R., and Song, X. (2019) Curcumin inhibits cell proliferation and migration in NSCLC through a synergistic effect on the TLR4/MyD88 and EGFR pathways. *Oncol Rep* **42**, 1843-1855

65. Cai, Y., Sheng, Z., and Liang, S. (2019) Radiosensitization effects of curcumin plus cisplatin on non-small cell lung cancer A549 cells. *Oncol Lett* **18**, 529-534

66. Somers-Edgar, T. J., Scandlyn, M. J., Stuart, E. C., Le Nedelec, M. J., Valentine, S. P., and Rosengren, R. J. (2008) The combination of epigallocatechin gallate and curcumin suppresses ER alpha-breast cancer cell growth in vitro and in vivo. *Int J Cancer* **122**, 1966-1971

67. Giommarelli, C., Zuco, V., Favini, E., Pisano, C., Dal Piaz, F., De Tommasi, N., and Zunino, F. (2010) The enhancement of antiproliferative and proapoptotic activity of HDAC inhibitors by curcumin is mediated by Hsp90 inhibition. *Cell Mol Life Sci* **67**, 995-1004

68. Gandhy, S. U., Kim, K., Larsen, L., Rosengren, R. J., and Safe, S. (2012) Curcumin and synthetic analogs induce reactive oxygen species and decreases specificity protein (Sp) transcription factors by targeting microRNAs. *BMC Cancer* **12**, 564

69. Jiang, X., and Huang, Y. (2020) Curcumin Derivative C086 Combined with Cisplatin Inhibits Proliferation of Osteosarcoma Cells. *Med Sci Monit* **26**, e924507

70. Yang, J., Zhu, D., Liu, S., Shao, M., Liu, Y., Li, A., Lv, Y., Huang, M., Lou, D., and Fan, Q. (2020) Curcumin enhances radiosensitization of nasopharyngeal carcinoma by regulating circRNA network. *Mol Carcinog* **59**, 202-214

71. Lee, J. Y., Lee, Y. M., Chang, G. C., Yu, S. L., Hsieh, W. Y., Chen, J. J., Chen, H. W., and Yang, P. C. (2011) Curcumin induces EGFR degradation in lung adenocarcinoma and modulates p38 activation in intestine: the versatile adjuvant for gefitinib therapy. *PLoS One* **6**, e23756

72. Zhou, J., Zhao, T., Ma, L., Liang, M., Guo, Y. J., and Zhao, L. M. (2017) Cucurbitacin B and SCH772984 exhibit synergistic anti-pancreatic cancer activities by suppressing EGFR, PI3K/Akt/mTOR, STAT3 and ERK signaling. *Oncotarget* **8**, 103167-103181

73. Klungsaeng, S., Kukongviriyapan, V., Prawan, A., Kongpetch, S., and Senggunprai, L. (2020) Targeted Modulation of FAK/PI3K/PDK1/AKT and FAK/p53 Pathways by Cucurbitacin B for the Antiproliferation Effect Against Human Cholangiocarcinoma Cells. *Am J Chin Med* **48**, 1475-1489

74. Gupta, P., and Srivastava, S. K. (2014) Inhibition of Integrin-HER2 signaling by Cucurbitacin B leads to in vitro and in vivo breast tumor growth suppression. *Oncotarget* **5**, 1812-1828

75. Baba, Y., Fujii, M., Maeda, T., Suzuki, A., Yuzawa, S., and Kato, Y. (2015) Deguelin Induces Apoptosis by Targeting Both EGFR-Akt and IGF1R-Akt Pathways in Head and Neck Squamous Cell Cancer Cell Lines. *BioMed Research International* **2015**, 1-9

76. Mehta, R., Katta, H., Alimirah, F., Patel, R., Murillo, G., Peng, X., Muzzio, M., and Mehta, R. G. (2013) Deguelin action involves c-Met and EGFR signaling pathways in triple negative breast cancer cells. *PLoS One* **8**, e65113

77. Farabegoli, F., Govoni, M., Spisni, E., and Papi, A. (2017) EGFR inhibition by (-)-epigallocatechin-3-gallate and IIF treatments reduces breast cancer cell invasion. *Biosci Rep* **37**

78. Hou, Z., Sang, S., You, H., Lee, M.-J., Hong, J., Chin, K.-V., and Yang, C. S. (2005) Mechanism of Action of (−)-Epigallocatechin-3-Gallate: Auto-oxidation–Dependent Inactivation of Epidermal Growth Factor Receptor and Direct Effects on Growth Inhibition in Human Esophageal Cancer KYSE 150 Cells. *Cancer Research* **65**, 8049-8056

79. Ma, Y. C., Li, C., Gao, F., Xu, Y., Jiang, Z. B., Liu, J. X., and Jin, L. Y. (2014) Epigallocatechin gallate inhibits the growth of human lung cancer by directly targeting the EGFR signaling pathway. *Oncol Rep* **31**, 1343-1349

80. Weng, L. X., Wang, G. H., Yao, H., Yu, M. F., and Lin, J. (2017) Epigallocatechin gallate inhibits the growth of salivary adenoid cystic carcinoma cells via the EGFR/Erk signal transduction pathway and the mitochondria apoptosis pathway. *Neoplasma* **64**, 563-570

81. Zhu, W., Li, M. C., Wang, F. R., Mackenzie, G. G., and Oteiza, P. I. (2020) The inhibitory effect of ECG and EGCG dimeric procyanidins on colorectal cancer cells growth is associated with their actions at lipid rafts and the inhibition of the epidermal growth factor receptor signaling. *Biochem Pharmacol* **175**, 113923

82. Cromie, M. M., Liu, Z., and Gao, W. (2017) Epigallocatechin-3-gallate augments the therapeutic effects of benzo[a]pyrene-mediated lung carcinogenesis. *Biofactors* **43**, 529-539

83. Singh, T., Gupta, N. A., Xu, S., Prasad, R., Velu, S. E., and Katiyar, S. K. (2015) Honokiol inhibits the growth of head and neck squamous cell carcinoma by targeting epidermal growth factor receptor. *Oncotarget* **6**, 21268-21282

84. Leeman-Neill, R. J., Cai, Q., Joyce, S. C., Thomas, S. M., Bhola, N. E., Neill, D. B., Arbiser, J. L., and Grandis, J. R. (2010) Honokiol inhibits epidermal growth factor receptor signaling and enhances the antitumor effects of epidermal growth factor receptor inhibitors. *Clin Cancer Res* **16**, 2571-2579

85. Wang, X., Beitler, J. J., Wang, H., Lee, M. J., Huang, W., Koenig, L., Nannapaneni, S., Amin, A. R., Bonner, M., Shin, H. J., Chen, Z. G., Arbiser, J. L., and Shin, D. M. (2014) Honokiol enhances paclitaxel efficacy in multi-drug resistant human cancer model through the induction of apoptosis. *PLoS One* **9**, e86369

86. Song, J. M., Anandharaj, A., Upadhyaya, P., Kirtane, A. R., Kim, J. H., Hong, K. H., Panyam, J., and Kassie, F. (2016) Honokiol suppresses lung tumorigenesis by targeting EGFR and its downstream effectors. *Oncotarget* **7**, 57752-57769

87. Fan, Y., Xue, W., Schachner, M., and Zhao, W. (2018) Honokiol Eliminates Glioma/Glioblastoma Stem Cell-Like Cells Via JAK-STAT3 Signaling and Inhibits Tumor Progression by Targeting Epidermal Growth Factor Receptor. *Cancers (Basel)* **11**

88. Yang, J., Pei, H., Luo, H., Fu, A., Yang, H., Hu, J., Zhao, C., Chai, L., Chen, X., Shao, X., Wang, C., Wu, W., Wan, L., Ye, H., Qiu, Q., Peng, A., Wei, Y., Yang, L., and Chen, L. (2017) Non-toxic dose of liposomal honokiol suppresses metastasis of hepatocellular carcinoma through destabilizing EGFR and inhibiting the downstream pathways. *Oncotarget* **8**, 915-932

89. Yang, J., Wu, W., Wen, J., Ye, H., Luo, H., Bai, P., Tang, M., Wang, F., Zheng, L., Yang, S., Li, W., Peng, A., Yang, L., Wan, L., and Chen, L. (2017) Liposomal honokiol induced lysosomal degradation of Hsp90 client proteins and protective autophagy in both gefitinib-sensitive and gefitinib-resistant NSCLC cells. *Biomaterials* **141**, 188-198

90. Dai, X., Li, R. Z., Jiang, Z. B., Wei, C. L., Luo, L. X., Yao, X. J., Li, G. P., and Leung, E. L. (2018) Honokiol Inhibits Proliferation, Invasion and Induces Apoptosis Through Targeting Lyn Kinase in Human Lung Adenocarcinoma Cells. *Front Pharmacol* **9**, 558

91. Okuda, K., Umemura, A., Umemura, S., Kataoka, S., Taketani, H., Seko, Y., Nishikawa, T., Yamaguchi, K., Moriguchi, M., Kanbara, Y., Arbiser, J. L., Shima, T., Okanoue, T., Karin, M., and Itoh, Y. (2021) Honokiol Prevents Non-Alcoholic Steatohepatitis-Induced Liver Cancer via EGFR Degradation through the Glucocorticoid Receptor-MIG6 Axis. *Cancers (Basel)* **13**

92. Gomathinayagam, R., Sowmyalakshmi, S., Mardhatillah, F., Kumar, R., Akbarsha, M. A., and Damodaran, C. (2008) Anticancer mechanism of plumbagin, a natural compound, on non-small cell lung cancer cells. *Anticancer Res* **28**, 785-792

93. Hafeez, B. B., Jamal, M. S., Fischer, J. W., Mustafa, A., and Verma, A. K. (2012) Plumbagin, a plant derived natural agent inhibits the growth of pancreatic cancer cells in in vitro and in vivo via targeting EGFR, Stat3 and NF-kappaB signaling pathways. *Int J Cancer* **131**, 2175-2186

94. Zhang, G. H., Cai, L. J., Wang, Y. F., Zhou, Y. H., An, Y. F., Liu, Y. C., Peng, Y., Chen, Z. F., and Liang, H. (2013) Novel compound PS-101 exhibits selective inhibition in non-small-cell lung cancer cell by blocking the EGFR-driven antiapoptotic pathway. *Biochem Pharmacol* **86**, 1721-1730

95. Jung, J. H., Lee, J. O., Kim, J. H., Lee, S. K., You, G. Y., Park, S. H., Park, J. M., Kim, E. K., Suh, P. G., An, J. K., and Kim, H. S. (2010) Quercetin suppresses HeLa cell viability via AMPK-induced HSP70 and EGFR down-regulation. *J Cell Physiol* **223**, 408-414

96. Bhat, F. A., Sharmila, G., Balakrishnan, S., Arunkumar, R., Elumalai, P., Suganya, S., Raja Singh, P., Srinivasan, N., and Arunakaran, J. (2014) Quercetin reverses EGF-induced epithelial to mesenchymal transition and invasiveness in prostate cancer (PC-3) cell line via EGFR/PI3K/Akt pathway. *J Nutr Biochem* **25**, 1132-1139

97. Balakrishnan, S., Mukherjee, S., Das, S., Bhat, F. A., Raja Singh, P., Patra, C. R., and Arunakaran, J. (2017) Gold nanoparticles-conjugated quercetin induces apoptosis via inhibition of EGFR/PI3K/Akt-mediated pathway in breast cancer cell lines (MCF-7 and MDA-MB-231). *Cell Biochem Funct* **35**, 217-231

98. Wang, G., Dai, F., Yu, K., Jia, Z., Zhang, A., Huang, Q., Kang, C., Jiang, H., and Pu, P. (2015) Resveratrol inhibits glioma cell growth via targeting oncogenic microRNAs and multiple signaling pathways. *Int J Oncol* **46**, 1739-1747

99. Jin, Z., Feng, W., Ji, Y., and Jin, L. (2017) Resveratrol mediates cell cycle arrest and cell death in human esophageal squamous cell carcinoma by directly targeting the EGFR signaling pathway. *Oncol Lett* **13**, 347-355

100. Chen, C. Y., Yu, Z. Y., Chuang, Y. S., Huang, R. M., and Wang, T. C. (2015) Sulforaphane attenuates EGFR signaling in NSCLC cells. *J Biomed Sci* **22**, 38

101. Pledgie-Tracy, A., Sobolewski, M. D., and Davidson, N. E. (2007) Sulforaphane induces cell type-specific apoptosis in human breast cancer cell lines. *Mol Cancer Ther* **6**, 1013-1021
